# Comparative efficacy of immune checkpoint inhibitors combined with chemotherapy in patients with advanced driver-gene negative non-small cell lung cancer: A systematic review and network meta-analysis

**DOI:** 10.1016/j.heliyon.2024.e30809

**Published:** 2024-05-07

**Authors:** Xuewen Zhang, Min Wu, Jie Chen, Kaiman Zheng, Huchen Du, Bo Li, Yujia Gu, Jun Jiang

**Affiliations:** a Department of Oncology, Graduate School of Qinghai University, Qinghai, China; b Department of Oncology, 903 Hosptial, Sichuan, China; c Division III, Department of Medical Oncology, Affiliated Hospital of Qinghai University, Qinghai, China

**Keywords:** NSCLC, Immune checkpoint inhibitor, Chemotherapy, First-line therapy, Network meta-analysis

## Abstract

**Objective:**

To evaluate the efficacy of different combinations of immune checkpoint inhibitors (ICIs) and chemotherapy (CT) in the treatment of advanced non-small cell lung cancer (NSCLC).

**Methods:**

We obtained relevant randomized controlled trials (RCTs) from databases such as PubMed, Embase, Web of Science, and The Cochrane Library up to May 31, 2023. The analysis of clinical prognostic factors was performed using R 4.2.3 and STATA 15.0. The main outcomes measured were overall survival (OS) and progression-free survival (PFS), while secondary outcomes included the objective response rate (ORR), disease control rate (DCR), and treatment-related adverse events of grade 3–5 severity (Grade ≥3 TRAE).

**Results:**

A total of 17 randomized controlled trials (RCTs) were conducted between 2012 and 2023, involving 7792 patients. These trials evaluated 11 different treatment methods. The results of these trials showed that in terms of overall survival (OS) and progression-free survival (PFS), the combination of tislelizumab with chemotherapy and the combination of camrelizumab with chemotherapy were particularly effective. Moreover, when compared with other combination therapies, pembrolizumab combined with chemotherapy showed superiority in terms of disease control rate (DCR) and objective response rate (ORR). Subgroup analyses further demonstrated that the addition of immune checkpoint inhibitors (ICIs) to chemotherapy significantly improved PFS and OS in patients without liver metastasis and in those with brain metastasis. Additionally, carboplatin-based combination therapy was found to confer favorable survival benefits in terms of PFS, while cisplatin-based combination therapy showed the most favorable outcomes in terms of OS. The results of subgroup analyses for overall survival (OS) showed that the combination of immunotherapy and chemotherapy yielded positive outcomes in specific subgroups. These subgroups were characterized by PD-L1 Tumor Proportion Score (TPS) of 50 % or higher, usage of anti-PD-1 medications, age below 65, male gender, smoking history, and non-squamous cell carcinoma histology. Superior effectiveness was demonstrated only in extending the progression-free survival (PFS) of female patients and patients with squamous carcinoma. Meanwhile, other patient cohorts did not show the same level of improvement.

**Conclusions:**

Tislelizumab, camrelizumab or pembrolizumab combined with chemotherapy may be the optimal first-line treatment strategies for NSCLC.

## Abbreviations

NSCLC: Non-small-cell lung cancer
EGFR: Epidermal growth factor receptor
ALK: Anaplastic lymphoma kinase
PD-1: Programmed cell death protein 1
PD-L1: Programmed cell death 1 ligand 1
CTLA-4: Cytotoxic T-lymphocyte-associated protein 4
ICIs: Immune checkpoint inhibitors
CT: Chemotherapy
TKIs: Tyrosine kinase inhibitors
RCT: Randomized controlled trial
PFS: Progression-free survival
OS: Overall survival
CR: Complete response
PR: Partial response
SD: Stable disease
PD: Progression disease
ORR: Objective response rate
DCR: Disease control rate
HR: Hazard Rate
CI: Confidence interval
PembroplusCT: Pembrolizumab + CT
SugeplusCT: Sugemalimab + CT
CamreplusCT: Camrelizumab + CT
AtezoplusCT: Atezolizumab + CT
TisleplusCT: Tislelizumab + CT
SintiplusCT: Sintilimab + CT
ToripplusCT: Toripalimab + CT
IpiplusCT: Ipilimumab + CT
NivoplusipiplusCT: Nivolumab + ipilimumab + CT
DurvaplustremeplusCT: Durvalumab + tremelimumab + CT
DurvaplusCT: Durvalumab + CT
NR: Not reported

## 1 Introduction

Lung carcinoma, a highly prevalent malignant tumor in clinical practice, remains the leading cause of cancer-related deaths globally [1]. Non-small-cell lung cancer (NSCLC) accounts for approximately 85 % of all lung cancer cases [2]. Unfortunately, NSCLC is challenging to detect in its early stages due to the absence of specific clinical manifestations, resulting in a vast majority (around 70 %) of patients receiving a diagnosis at an advanced stage. Consequently, the 5-year survival rate for these patients is only 15 %–17 % [3]. In recent years, targeted therapies have emerged as a breakthrough in the treatment of NSCLC. These therapies have shown remarkable benefits for approximately 30–40 % of NSCLC patients who possess sensitive mutations in the epidermal growth factor receptor (EGFR) or anaplastic lymphoma kinase (ALK). Precision-targeted treatments, such as EGFR-TKI and ALK-TKI, have now become the standard first-line therapy for these patients [4,5]. However, for patients with locally advanced or metastatic NSCLC who do not possess driver gene mutations, their treatment options are still limited to platinum-based cytotoxic chemotherapy. While this approach does provide an extension of survival, it inevitably comes with moderate-to-severe toxicities and side effects [6].

The immune system has crucial pathways called immune checkpoint pathways. These pathways, such as programmed cell death protein 1 (PD-1)/programmed cell death 1 ligand 1 (PD-L1) and cytotoxic T-lymphocyte-associated protein 4 (CTLA-4), play a role in immunosuppression and evasion of the immune system by malignant tumors. To counteract this, immune checkpoint inhibitors (ICIs) have been developed. ICIs are monoclonal antibody analogs that target these immune checkpoints, leading to the reactivation of anti-tumor immune responses and resulting in effective anti-tumor effects [7]. Numerous phase III randomized controlled trials have been conducted, demonstrating the significant advantages of ICIs over chemotherapy in patients with NSCLC. The results from the KEYNOTE-024 trial (NCT02142738) demonstrated a significant improvement in overall survival (OS) for patients treated with pembrolizumab. The pembrolizumab group had a median OS of 26.3 months (95 % CI, 18.3–40.4), compared to 13.4 months (95 % CI, 9.4–18.3) for patients in the chemotherapy group (hazard ratio [HR] = 0.62; 95 % CI, 0.48–0.81). It is noteworthy that grade 3/4 immune-related adverse events (IRAEs) associated with pembrolizumab treatment were observed in 9.7 % of patients, with the most common sites being the lung (2.6 %), skin (3.9 %), and gastrointestinal tract (1.3 %) [8]. The results from the KEYNOTE-042 trial (NCT02220894) revealed that among three stratified populations with different tumor proportion scores (TPS) (PD-L1 ≥50 %, ≥20 %, and ≥1 %), the pembrolizumab group (PD-L1 ≥ 50 % strata) demonstrated a significantly longer overall survival (OS) (HR 0.69, 95 % CI: [0.56, 0.85]) and a much lower incidence of Grade ≥3 treatment-related adverse events (18 % in the pembrolizumab group vs. 41 % in the chemotherapy group) compared to the chemotherapy group. In the EMPOWER-Lung 01 study (NCT03088540) [9], cemiplimab monotherapy was found to improve the progression-free survival (PFS) of patients with advanced non-small cell lung cancer (NSCLC) who had PD-L1 ≥ 50 % (median PFS: 8.2 months vs. 5.7 months; HR 0.54, 95 % CI: [0.43, 0.68]). However, Darvin et al. [10] concluded that only 50 % of patients would benefit from immune checkpoint inhibitor (ICI) monotherapy, possibly due to the lack of significant survival benefit in the group with PD-L1 expression levels ≥1 % or ≥20 %, as demonstrated in the KEYNOTE-042 trial [11]. On the other hand, the JAVELIN Lung 100 trial [12] did not establish, through an independent review committee (IRC), that avelumab is superior to platinum-based dual-drug chemotherapy in terms of OS or PFS in the first-line treatment of patients with high-expression PD-L1-positive tumors. Carbone et al. [13] also concluded that nivolumab alone did not improve survival in patients with stage IV NSCLC who had PD-L1 ≥ 5 % when compared to chemotherapy.

Recent studies have revealed that the integration of immune checkpoint inhibitors (ICIs) into first-line chemotherapy regimens for patients with advanced non-small cell lung cancer (NSCLC) has shown promising results in terms of improving survival rates, while also managing toxic side effects effectively [14]. Theoretically, chemotherapy has the potential to enhance the recognition of T cells towards neoantigens, thereby increasing the immunogenicity of the tumor. At the same time, immunotherapy works by inhibiting T-cell checkpoints, leading to a strengthened anti-tumor immune response. When combined, these two treatment modalities exhibit a synergistic effect [15]. As a result, the combination of immunotherapy and chemotherapy acts synergistically to enhance the effectiveness of PD-1 and PD-L1 inhibitors in combating cancer. Hence, it is important to note that the advent of immune checkpoint inhibitors does not replace conventional therapeutic paradigms, but rather complements the existing treatment options available.

The pivotal trial, KEYNOTE-189, which is a Phase 3 randomized controlled trial, marked the beginning of the era of combination immunotherapy treatment for non-small cell lung cancer (NSCLC). The findings revealed an objective response rate of 86.0 % among the 57 patients who received 35 cycles of pembrolizumab therapy. Moreover, a 3-year overall survival (OS) rate of 71.9 % was recorded approximately 5 years after randomization. Notably, the advantage of pembrolizumab-pemetrexed-platinum chemotherapy combination over placebo-pemetrexed-platinum group was sustained in terms of both OS and progression-free survival (PFS), regardless of PD-L1 expression levels [16]. This research strongly suggests that incorporating immune checkpoint inhibitors (ICIs) into first-line chemotherapy regimens for advanced NSCLC patients can significantly improve their survival rates. Currently, the use of immunotherapy-chemotherapy combination approach has been approved for both squamous and non-squamous cell carcinomas, irrespective of PD-1/PD-L1 expression levels [17].

However, the optimal treatment regimen for advanced non-small cell lung cancer (NSCLC) without driver gene mutations remains unclear, despite the availability of numerous immune checkpoint inhibitors and chemotherapy drugs that can be combined. To address this issue, a network meta-analysis is currently underway, utilizing existing randomized controlled trials. Its objective is to investigate the relative effectiveness and safety of first-line treatments using immunotherapy combined with chemotherapy (ICI + CT) or dual immunotherapy combined with chemotherapy (ICI + ICI + CT). Furthermore, subsequent subgroup analyses will be conducted based on factors including organ metastasis and specific chemotherapy drugs. These analyses aim to identify the most effective and safe treatment approach for patients in different clinical scenarios.

## 2 Materials and methods

### 2.1 Literature retrieval

The study followed the guidelines recommended by the Preferred Reporting Items for Programmatic Systematic Reviews and Meta-Analyses (PRISMA-P) [18]. Within this framework, two commonly used network meta-analysis models, Frequency theory and Bayesian theory, were examined. The Bayesian network meta-analysis model was selected for its ability to handle complex situations [19]. Moreover, the study has been registered in the International Prospective Register of Systematic Reviews with the PROSPERO ID CRD42023487239.

A comprehensive literature search was conducted on May 31, 2023, using the PubMed, Embase, Web of Science, and the Cochrane Library databases. The search focused on the keywords “immune checkpoint inhibitors,” “non-small cell lung cancer,” “chemotherapy,” and “randomized controlled trials.” The supplementary material 1 provides a detailed outline of the search strategy.

### 2.2 Literature screening

The study's inclusion criteria were as follows: (1) Patient population: individuals diagnosed with histologically or cytologically confirmed advanced non-small cell lung cancer (NSCLC) [20]; (2) Intervention: phase II or III randomized controlled trials evaluating the first-line treatment of immune checkpoint inhibitors combined with chemotherapy (ICI + CT) or dual immune checkpoint inhibitors combined with chemotherapy (ICI + ICI + CT) [5]; (3) Outcome measures: included studies reporting one or more of the following outcomes: a. Progression-free survival (PFS), defined as the time from randomization to the first occurrence of disease progression (local or distant) or death; b. Overall survival (OS), defined as the time from randomization to death from any cause; c. Objective remission rate (ORR), defined as the proportion of patients achieving objective remission; d. Disease control rate (DCR), defined as the proportion of patients achieving complete remission (CR), partial remission (PR), or stable disease (SD); and e. Grade ≥3 treatment-related adverse events (TRAEs) or specific adverse events graded according to the National Cancer Institute Common Terminology Criteria for Adverse Events (NCI-CTCAE).

The exclusion criteria were applied as follows: (1) Non-randomized phase II studies and phase I studies were excluded. (2) Studies were excluded if patients underwent immunotherapy or received treatment modalities other than chemotherapy as initial treatment. (3) Studies involving patients with mutations sensitive to epidermal growth factor receptor, mesenchymal lymphoma kinase, or other genetic mutations were also excluded. (4) Studies where the efficacy of the drug could not be determined were excluded. (5) Duplicate literature, systematic reviews, case reports, meta-analyses, letters, or non-English literature were also excluded. In addition, the references of the included trials were thoroughly reviewed to ensure that all eligible studies were accounted for. In cases where multiple articles reported on the same clinical trial, the most recently published or fully reported literature was selected. Alternatively, both articles were considered to complement each other. Any disputes regarding the studies were resolved through adjudication by a third investigator (WM).

### 2.3 Data extraction and quality assessment

All articles meeting the inclusion criteria were independently examined by two investigators (ZXW, WM). A spreadsheet was utilized to record the trial details, including the author, year of publication, phase, intervention, sample size, histologic type, gender and age, tobacco use, PD-L1 expression, presence of liver metastases, presence of brain metastases, median follow-up time, and primary outcome indicators such as overall survival (OS), progression-free survival (PFS), objective response rate (ORR), disease control rate (DCR), and treatment-related adverse events (TRAE). During the data extraction process, utmost importance was given to retrieving survival data evaluated by an independent review committee in order to prevent potential biases in the evaluation among the investigators. Additionally, any studies with three arms (such as RATIONALE-307 and POSEIDON) [21,22] were individually classified and labeled due to the presence of two-by-two comparisons, ensuring the integrity of the study.

We utilized the Cochrane bias risk tool to evaluate the bias risk of each study [23]. Our quality assessment focused primarily on seven key aspects: (1) generation of randomized sequences; (2) allocation concealment; (3) blinding of patients and investigators; (4) blinding of outcome assessors; (5) incompleteness of outcome data; (6) selective outcome reporting; and (7) other potential sources of bias. For each aspect, we classified the risk as unclear (yellow), low (green), or high (red). The evaluation of bias risk for each study was conducted independently by two investigators (WM and ZXW), with any discrepancies resolved through a review panel (ZXW, WM, JJ, CJ, and ZKM).

### 2.4 Data synthesis and statistical analysis

To assess the efficacy and safety of different treatment options, a comprehensive analysis was conducted by synthesizing all available direct and indirect evidence. Hazard ratios (HR) were utilized to measure progression-free survival (PFS) and overall survival (OS), while odds ratios (OR) were employed to present the objective response rate (ORR), disease control rate (DCR), and the occurrence of grade ≥3 treatment-related adverse events (TRAE). Additionally, the corresponding 95 % confidence intervals were provided. The primary endpoints focused on OS and PFS, while the secondary endpoints included ORR, DCR, and the occurrence of grade ≥3 TRAE.

Stata 15.0 was utilized to generate network diagrams representing various treatment regimens for different outcomes. These diagrams were employed to ascertain whether the treatment regimens in the included studies were compared directly or indirectly [24]. The forest plot was used to assess heterogeneity between studies, employing the I^2^ statistic. Heterogeneity levels were categorized as low (<25 %), moderate (between 25 % and 50 %), or high (>50 %) [25]. We performed a network meta-analysis on advanced NSCLC patients without driver gene mutations, including subgroup analysis. To achieve this, we utilized Markov Chain Monte Carlo simulation within a Bayesian framework. Our approach incorporated the use of GeMTC, a valuable tool in this analysis. The GeMTC software was configured with 20,000 tuning iterations and 50,000 simulating iterations [26,27]. To determine the appropriate model (random-effects, fixed-effects, or consistent/inconsistent-effects) for the analysis, I^2^ and DIC values were considered. In order to evaluate the model's convergence, we used the potential scale reduction factor (PSRF) [28] based on the Brooks-Gelman-Rubin method. A PSRF value closer to 1 indicates better convergence. In the Bayesian network meta-analysis, treatment modalities were ranked using the area under the cumulative ranking curve. This curve ranges from 0 (worst treatment) to 1 (best treatment) [28]. Publication bias was assessed by generating funnel plots using Stata 15 after formatting the data. Since the network graph does not form a closed loop, the node-splitting method was not employed.

## 3 Results

### 3.1 Literature search

**Fig. 1:**
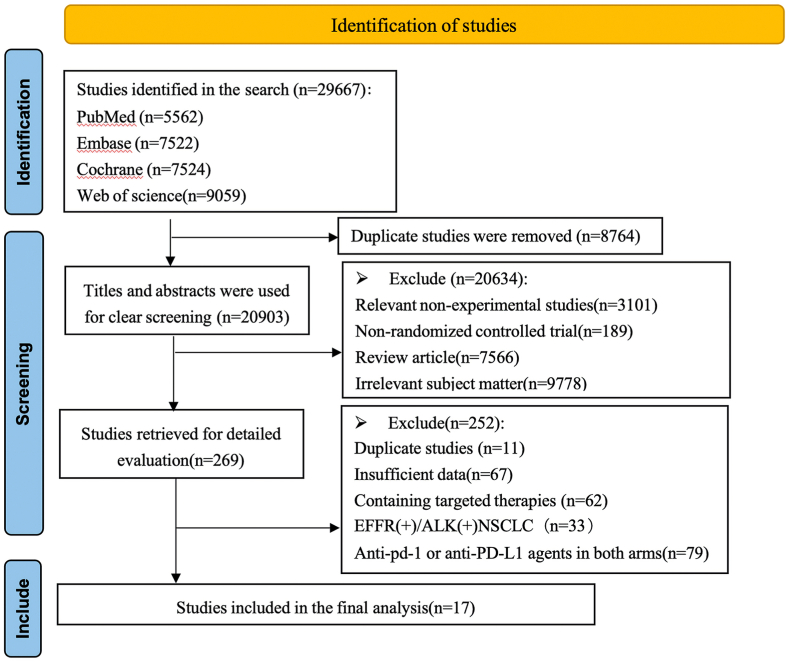
Flow diagram representing the selection process.

Based on the initial search, a total of 29,667 articles were identified as relevant. After eliminating 8764 duplicates, an additional 20,634 articles were excluded based on the predetermined inclusion criteria. Subsequently, the complete texts of the remaining 269 studies that were potentially eligible underwent comprehensive screening. As a result, 17 randomized controlled trials were found to meet the criteria and were included in the final analysis (Fig. 1).

### 3.2 Study characteristics

This study analyzed a cohort of 7792 patients and investigated 11 different treatment approaches. Among the literature reviewed, there were 15 Phase III studies [16,21,22,[29], [30], [31], [32], [33], [34], [35], [36], [37], [38], [39], [40]] and 2 Phase II studies [41,42]. All participants were adult patients with a confirmed diagnosis of advanced or metastatic non-small cell lung cancer (NSCLC). Specifically, 5 studies focused on patients with squamous cell carcinoma [22,33,36,38,40], 7 on patients with non-squamous cell carcinoma [16,21,29,34,35,37,41], and 5 on patients with histologic NSCLC [[30], [31], [32],^39^,^42^]. The initial treatment for all patients consisted of immunotherapy in combination with chemotherapy. The most frequently used combination regimen among the included studies was Pembrolizumab (n = 4). Among the 6060 patients who received immune-monotherapy in combination with chemotherapy, Pembrolizumab-based treatment had the highest number of cases (773), while atezolizumab had the lowest (292). Additionally, 1732 patients received dual immunotherapy (nivolumab+ipilimumab/durvalumab + toripalimab) in conjunction with chemotherapy. The other treatment protocols utilized in the 17 studies were as follows: tislelizumab + chemotherapy in 2 studies [21,40], atezolizumab + chemotherapy in 1 study [34], ipilimumab + chemotherapy in 2 studies [33,42], camrelizumab + chemotherapy in 2 studies [22,29], sintilimab + chemotherapy in 2 studies [37,38], sugemalimab + chemotherapy in 1 study [32], toripalimab + chemotherapy in 1 study [31], durvalumab + chemotherapy in 1 study [39], nivolumab + ipilimumab + chemotherapy in 1 study [30], and durvalumab + toripalimab + chemotherapy in 1 study [39]. A summary of the key characteristics of the included studies can be found in Table 1.

**Table 1:** Baseline characteristics of studies.

NO	Study (year)	Phase	N	stage	E/C	Network comparator (s)	Histology, n (%)	PFS, HR (95%CI)	OS, HR (95%CI)	ORR, E/C (%)	Grade ≥3 TRAEs, E/C (%)	DCR	Median follow up (months)
1	Awad et al. (2021), KEYNOTE-021	2	123	IIIB/IV	60	**Pembrolizumab + CT** (pemetrexed + platinum)	non-squamous: 60 (100)	0.54 (0.35–0.83)	0.71(0.45–1.12)	35(58)	23(39)	53(88)	49.4
					63	**CT** (placebo + pemetrexed + platinum)	non-squamous: 63 (100)			21(33)	20(31)	44(70)	
2	Zhou et al. (2022), GEMSTONE-302	3	479	IV	320	**Sugemalimab + CT** (carboplatin + paclitaxel)	squamous：129(40)	0.48 (0.39–0.60)	0.65 (0.500.84)	203(63.4)	205(64)	NR	17.8
						**Sugemalimab + CT** (carboplatin + pemetrexed)	non-squamous：191(60)					NR	
					159	**CT** (placebo + carboplatin + paclitaxel)	squamous：63 (40)			68(40.3)	99(62)	NR	
						**CT** (placebo + carboplatin + pemetrexed)	non-squamous：96 (60)					NR	
3	Zhou et al. (2021), CameL	3	412	IIIB–IV	205	**Camrelizumab + CT** (carboplatin + pemetrexed)	non-squamous: 205(100)	0.55 (0.44–0.69)	0.72(0.57–0.92)	113(55.1)	145(70.7)	180(87.8)	24.2
					207	**CT** (carboplatin + pemetrexed)	non-squamous: 207(100)			68(32.9)	101(48.8)	154(74.4)	
4	Ren et al. (2021), CameL-sq	3	389	IIIB-IV	193	**Camrelizumab + CT** (carboplatin + paclitaxel)	squamous：193(100)	0.37 (0.29–0.47)	0.55 (0.40–0.75)	125(64.8)	143(74)	176(91)	13.5
					196	**CT (placebo + carboplatin + paclitaxe)**	squamous：196(100)			72(36.7)	141(72)	178(91)	
5	Abreu et al. (2021), KEYNOTE-189	3	616	IV	410	**Pembrolizumab + CT** (pemetrexed + cisplatin/carboplatin)	non-squamous: 410(100)	0.49 (0.41–0.59)	0.56(0.46–0.69)	198(48.3)	292(72.1)	347(85)	31
					206	**CT** (placebo + pemetrexed + cisplatin/carboplatin)	non-squamous: 206(100)			41(19.9)	135(66.8)	145(70)	
6	Nishio et al. (2020), IMpower132	3	578	IV	292	**Atezolizumab + CT** (pemetrexed + carboplatin or cisplatin)	non-squamous：292(100)	0.56 (0.47–0.67)	0.86(0.71–1.06)	137(47)	171(58.4)	NR	28.4
					286	**CT** (pemetrexed + carboplatin or cisplatin)	non-squamous：286(100)			92(32)	123(43)	NR	
7	Novello et al. (2023), KEYNOTE-407	3	559	IV	278	**Pembrolizumab + CT** (carboplatin + paclitaxel)	squamous：278(100)	0.57 (0.47–0.69)	0.71(0.58–0.88)	174(62.6)	208(74.8)	239(86)	56.9
					281	**CT** (placebo + carboplatin + paclitaxel)	squamous：281(100)			108(38.4)	197(70)	211(75)	
8	Lu et al. (2021), RATIONALE 304	3	332	IIIB–IV	222	**Tislelizumab + CT** (platinum + pemetrexed)	non-squamous：222(100)	0.63 (0.47–0.86)	0.68 (0.50, 0.92)	127(57.4)	150(67.6)	198(89.2)	38.8
					110	**CT** (platinum and pemetrexed)	non-squamous：110(100)			41(36.9)	59(53.6)	89(81.1)	
9	Wang et al. (2021), RATIONALE-307	3	360	IIIB–IV	120	**Tislelizumab + CT** (paclitaxel + carboplatin)	squamous：120(100)	0.45 (0.33, 0.62) AC	0.53(0.38–0.75)	87(72.5)	103(85.8)	106(88)	39.8
					119	**Tislelizumab + CT** (nab-paclitaxel + carboplatin)	squamous：119(100)	0.43 (0.31. 0.60) BC	0.60(0.43–0.83)	89(74.8)	99(83.9)	108(91)	
					121	**CT** (paclitaxel + carboplatin)	squamous：121(100)			60(49.6)	94(80.3)	97(80)	
10	Zhang et al. (2022),ORIENT-11	3	397	IIIB–IV	266	**Sintilimab + CT** (pemetrexed + platinum)	non-squamous：266(100)	0.49 (0.38, 0.63)	0.65(0.50,0.85)	138(51.9)	164(61.7)	231(86.8)	30.8
					131	**CT** (placebo + pemetrexed + platinum)	non-squamous：131(100)			39(29.8)	77(58.8)	99(75.6)	
11	Zhou et al. (2021), ORIENT-12	3	357	IIIB–IV	179	**Sintilimab + CT** (platinum + gemcitabine)	squamous：179(100)	0.536(0.422–0.681)	0.567(0.353–0.909)	80(44.7)	155(86.6)	NR	12.9
					178	**CT** (placebo + platinum + gemcitabine)	squamous：178(100)			63(35.4)	148(83.1)	NR	
12	Wang et al. (2023), CHOICE-01	3	465	IIIB–IV	309	**Toripalimab + CT** (nab-paclitaxel + carboplatin)	squamous：147(48)	0.49 (0.39–0.61)	0.69(0.53–0.92)	203(65.7)	243(78.6)	NR	7.1
						**Toripalimab + CT** (pemetrexed + cisplatin/carboplatin)	non-squamous：162(52)						
					156	**CT** (placebo + nabpaclitaxel + carboplatin)	squamous：73(47)			72(46.2)	128(82.1)	NR	
						**CT** (placebo + pemetrexed + cisplatin/carboplatin)	non-squamous：83(53)						
13	Lynch et al., 2012	2	204	IIIB–IV	70	**Ipilimumab + CT** (Ipilimumab + paclitaxel + carboplatin followed by placebo + paclitaxel + carboplatin)	NSCLC：70(100)	0.81 (0.55–1.17) AC	0.99 (0.67–1.46) AC	15(21)	29(41)	40(57)	NR
					68	**Ipilimumab + CT** (Placebo + paclitaxel + arboplatin followed by ipilimumab + paclitaxel + carboplatin)	NSCLC：68(100)	0.72 (0.50–1.06) BC	0.87 (0.59–1.28) BC	15(21)	27(39)	53(78)	
					66	**CT** (Placebo + paclitaxel + carboplatin)	NSCLC：66(100)			9(14)	24(37)	48(73)	
14	Govindan R, (2017), NCT01285609	3	749	IV	388	**Ipilimumab + CT** (paclitaxel + carboplatin)	squamous：388(100)	0.87 (0.75–1.01)	0.91(0.77–1.07)	171(44)	206(53)	314(81)	12.5
					361	**CT** (placebo + paclitaxel + carboplatin)	squamous：361(100)			170(47)	130(36)	319(88)	
15	Ares et al. (2021), CheckMate 9LA	3	719	IV	361	**Nivolumab + Ipilimumab + CT** (pemetrexed + platinum(non-squamous/carboplatin + paclitaxel(squamous)	NSCLC: 361(100)	0.67 (0.56–0.79)	0.72(0.61–0.86)	137(38)	168(47)	302(84)	30.7
					358	**CT** (pemetrexed + platinum(non-squamous)/carboplatin + paclitaxel(squamous))	NSCLC: 358(100)			91(25.4)	132(38)	274(77)	
16	Johnson et al. (2022), POSEIDON	3	1013	IV	338	**Durvalumab + Tremelimumab + CT**	NSCLC：338(100)	0.72 (0.60–0.86) AC	0.77(0.65–0.92) AC	131(38.8)	175(51.8)	NR	34.9
					338	**Durvalumab + CT**	NSCLC：338(100)	0.74 (0.62–0.89) BC	0.86(0.72–1.02) BC	140(41.5)	151(44.6)	NR	
					337	**CT** (gemcitabine + carboplatin/cisplatin(squamous), pemetrexed+ carboplatin/cisplatin (non-squamous), or nab-paclitaxel + carboplatin (either))	NSCLC：337(100)			82(24.4)	150(44.4)	NR	
17	Horinouchi et al. (2021), KEYNOTE-189 Japan Study	3	40	Ⅳ	25	**Pembrolizumab + CT** (pemetrexed + platinum)	non-squamous：25(100)	0.62 (0.27–1.42)	0.29(0.07–1.15)	14(56)	18(72)	23(92)	18.5
					15	**CT** (placebo + pemetrexed + platinum)	non-squamous：15(100)			5(33)	9 (60)	13(87)	

### 3.3 Risk-of-bias assessments

**Fig. 2:**
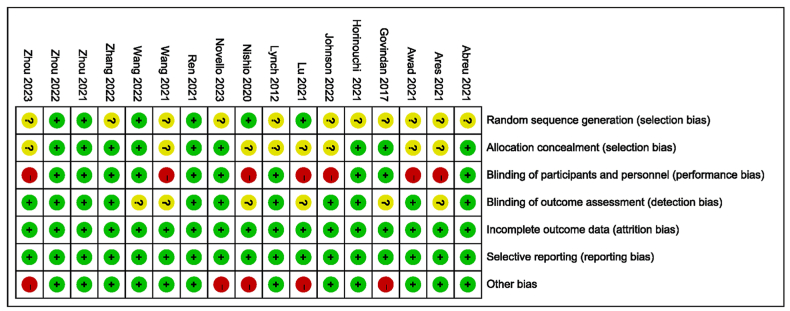
Assessment of bias of included studies using Cochrane Collaboration risk of bias assessment tool.

All studies (Fig. 2) demonstrated a low risk of bias. Seven studies [21,29,30,34,[39], [40], [41]] were identified as having a high risk of implementation bias due to their open-label design, which resulted in a lack of blinding. Five studies [21,29,33,34,36] exhibited a high risk of other bias due to significant discontinuation rates. Furthermore, an uncertain risk of bias was observed in other areas such as the methodological reporting of randomized sequence generation, allocation concealment, and blinding of outcome assessors due to inadequate descriptions of these components.

### 3.4 Therapeutic effect and safety

We conducted a meta-analysis to evaluate the effectiveness of various treatment therapies. Seventeen studies, with a total of 7792 patients, examined parameters such as PFS, OS, ORR, and DCR. The forest plot of the network meta-analysis revealed that immunotherapy combination treatments significantly improved the OS, PFS, ORR, and DCR of patients compared to chemotherapy. These findings were statistically significant.

#### 3.4.1 PFS, OS

**Fig. 3:**
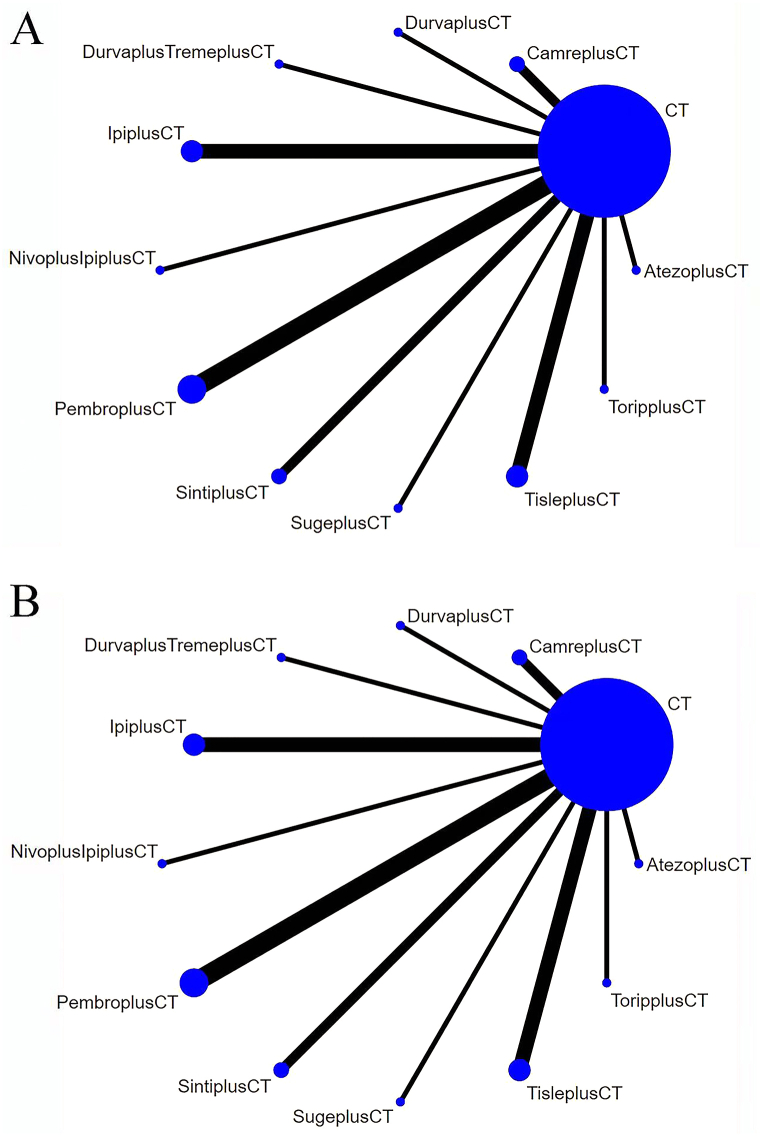
Network Diagram. **A:** Network diagram of OS; **B:** Network diagram of PFS. Circular nodes represent treatment regimens. The size of each circle indicates the number of cases. The width of the lines indicates the number of studies. CT = chemotherapy. PembroplusCT = pembrolizumab + CT. SugeplusCT = sugemalimab + CT. CamreplusCT = camrelizumab + CT. AtezoplusCT = Atezolizumab + CT. TisleplusCT = tislelizumab + CT. SintiplusCT = sintilimab + CT. ToripplusCT = toripalimab + CT. IpiplusCT = ipilimumab + CT. NivoplusipiplusCT = nivolumab + ipilimumab + CT. DurvaplustremeplusCT = durvalumab + tremelimumab + CT. DurvaplusCT = durvalumab + CT

**Fig. 4:**
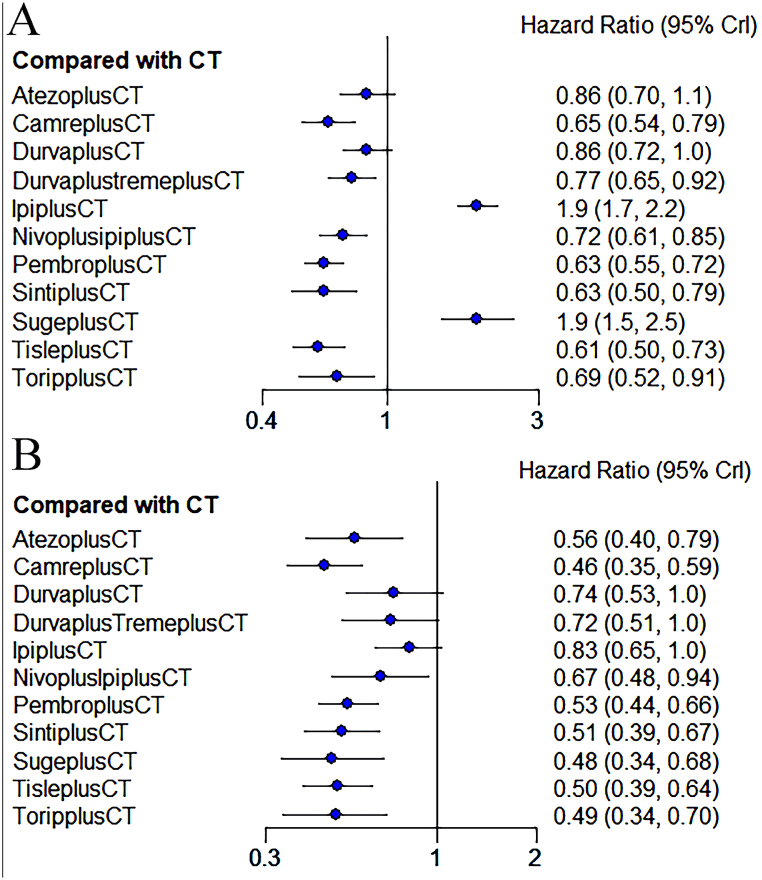
Forest plot of comparison of OS **(A)** and PFS **(B)**. Forest plot comparing overall survival (OS) and progression-free survival (PFS) between immunotherapy and chemotherapy. The treatments included in the analysis are as follows: Pembrolizumab + CT (chemotherapy) = PembroplusCT- Sugemalimab + CT = SugeplusCT- Camrelizumab + CT = CamreplusCT- Atezolizumab + CT = AtezoplusCT- Tislelizumab + CT = TisleplusCT- Sintilimab + CT = SintiplusCT- Toripalimab + CT = ToripplusCT- Ipilimumab + CT = IpiplusCT- Nivolumab + Ipilimumab + CT = NivoplusipiplusCT- Durvalumab + Tremelimumab + CT = DurvaplustremeplusCT- Durvalumab + CT = DurvaplusCT

The efficacy of 11 different treatment modalities was compared in this study. Comparative network plots are illustrated in Fig. 3A and B. The forest plot for OS (Fig. 4A) indicated that tislelizumab + chemotherapy showed the highest effectiveness in improving OS compared to the other treatments, with a hazard ratio (HR) of 0.61 and a 95 % confidence interval (CI) of [0.50, 0.73]. Several other treatments also exhibited superior effectiveness in improving OS compared to chemotherapy alone. These include camrelizumab (HR 0.65, 95 % CI: [0.54, 0.79]), durvalumab + toripalimab + chemotherapy (HR 0.77, 95 % CI: [0.65, 0.92]), nivolumab + ipilimumab + chemotherapy (HR 0.72, 95 % CI: [0.61, 0.85]), pembrolizumab + chemotherapy (HR 0.63, 95 % CI: [0.55, 0.72]), sintilimab + chemotherapy (HR 0.63, 95 % CI: [0.50, 0.79]), and toripalimab + chemotherapy (HR 0.69, 95 % CI: [0.52, 0.91]). All these differences were statistically significant. Atezolizumab + chemotherapy (HR 0.86, 95 % CI: [0.70, 1.1]) and durvalumab + chemotherapy (HR 0.86, 95 % CI: [0.72, 1.0]) also showed superiority to chemotherapy alone, although the difference was not statistically significant. However, ipilimumab + chemotherapy (HR 1.9, 95 % CI: [1.7, 2.2]) and sugemalimab + chemotherapy (HR 1.9, 95 % CI: [1.5, 2.5]) were found to be inferior to chemotherapy alone.

Similarly, as shown in the forest plot for progression-free survival (PFS) (Fig. 4B), the combination of camrelizumab with chemotherapy (0.46, [0.35, 0.59]) demonstrated superior efficacy compared to other combination therapies in enhancing PFS. These include atezolizumab combined with chemotherapy (0.56, [0.40,0.79]), nivolumab combined with ipilimumab and chemotherapy (0.67, [0.48, 0.94]), pembrolizumab combined with chemotherapy (0.53, [0.44, 0.66]), sintilimab combined with chemotherapy (0.51, [0.39, 0.67]), sugemalimab combined with chemotherapy (0.48, [0.34, 0.68]), tislelizumab combined with chemotherapy (0.50, [0.39, 0.64]), and toripalimab combined with chemotherapy (0.49, [0.34, 0.70]). Furthermore, the efficacy of other therapies such as durvalumab combined with toripalimab and chemotherapy (0.72, [0.51, 1.0]), durvalumab combined with chemotherapy (0.74, [0.53, 1.0]), and ipilimumab combined with chemotherapy (0.83, [0.65, 1.0]) did not show statistically significant differences from that of chemotherapy alone. The comparison between immunotherapy and chemotherapy in terms of the survival of patients with advanced non-small cell lung cancer (NSCLC) was subsequently conducted using league tables (Table 2). In terms of improving overall survival (OS), camrelizumab combined with atezolizumab and chemotherapy (1.32, [1, 1.74]), durvalumab combined with toripalimab and chemotherapy (0.85, [0.66, 1.1]), nivolumab combined with ipilimumab and chemotherapy (0.91, [0.7, 1.17]), pembrolizumab combined with chemotherapy (1.03, [0.82, 1.31]), sintilimab combined with chemotherapy (1.04, [0.77, 1.4]), tislelizumab combined with chemotherapy (1.08, [0.82,1.4]), and toripalimab combined with chemotherapy (0.95, [0.68, 1.32]) exhibited similar efficacy, as the hazard ratios (HRs) were close to 1. In terms of improving PFS, the combination of dual immunotherapy with chemotherapy (DurvaplusTremeplusCT, NivoplusIpiplusCT) and the combination of single immunotherapy with chemotherapy (DurvaplusCT, IpiplusCT) did not display significant differences in efficacy ((0.97, [0.6, 1.57]), (0.81, [0.55, 1.24])). However, the single immunotherapy IpiplusCT was found to be more effective in prolonging OS compared to the dual immunotherapy NivoplusipiplusCT (0.38, [0.3, 0.47]).

**Table 2:**
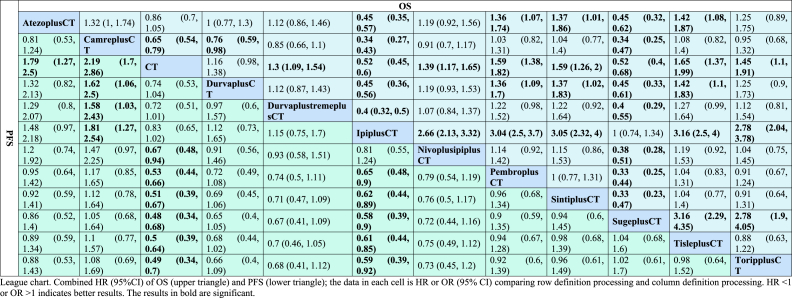
League chart of OS and PFS.

**Fig. 5:**
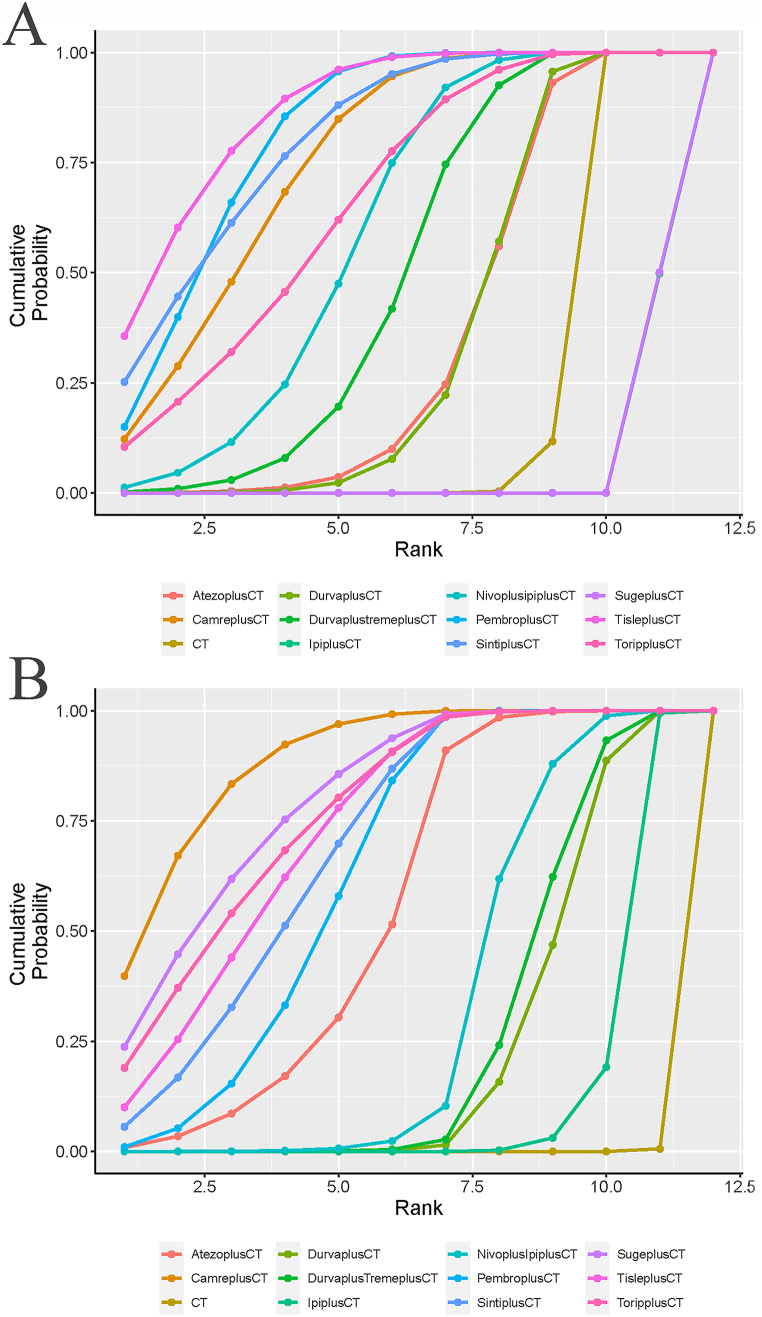
Ranking diagram of OS **(A)** and PFS **(B)**. PembroplusCT = pembrolizumab + CT; SugeplusCT = sugemalimab + CT; CamreplusCT = camrelizumab + CT; AtezoplusCT = Atezolizumab + CT; TisleplusCT = tislelizumab + CT; SintiplusCT = sintilimab + CT; ToripplusCT = toripalimab + CT; IpiplusCT = ipilimumab + CT; NivoplusipiplusCT = nivolumab + ipilimumab + CT; DurvaplustremeplusCT = durvalumab + tremelimumab + CT; DurvaplusCT = durvalumab + CT

The cumulative rankings plot demonstrates that tislelizumab achieved the highest position in terms of overall survival (OS) improvement among non-small cell lung cancer (NSCLC) patients, with a SUCRA value of 87.1 % (Fig. 5A). Following closely behind are pembrolizumab (81.9 %) and sintilimab (80.7 %). However, sugemalimab and ipilimumab ranked lower than chemotherapy, with chemotherapy scoring 19.2 %, sugemalimab scoring 4.55 %, and ipilimumab scoring 4.53 % respectively, placing them in the last three positions. In terms of progression-free survival (PFS) improvement (Fig. 5B), camrelizumab is most likely to secure the top ranking, with a score of 88.9 %. It is followed by sugemalimab (80.3 %) and toripalimab (70.0 %). Durvalumab (23.0 %), ipilimumab (11.0 %), and chemotherapy (0.53 %) are ranked at the bottom.

#### 3.4.2 ORR, DCR

**Fig. 6:**
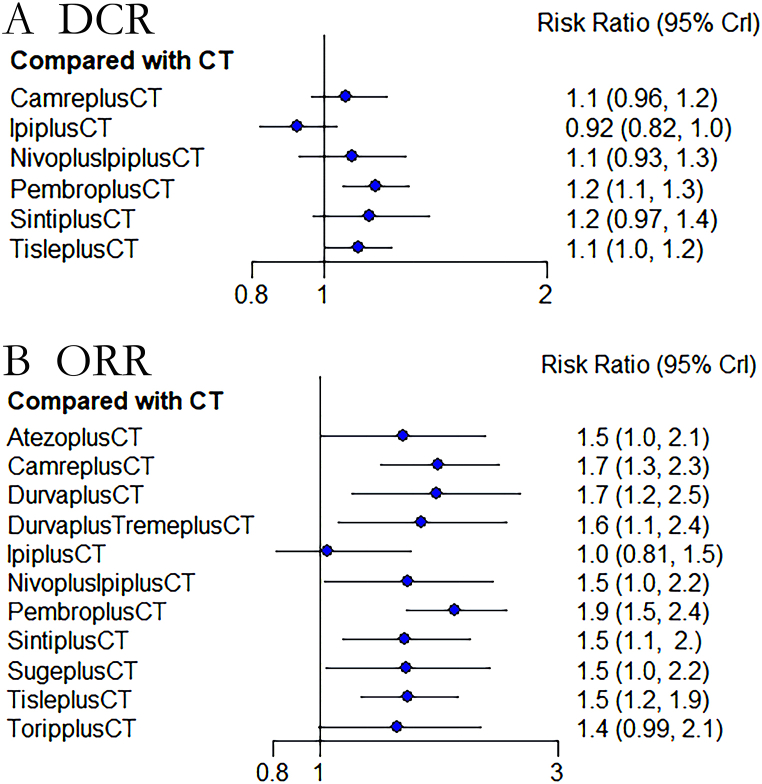
Forest plot of DCR**(A)** and ORR **(B)**. Forest plot comparing the Disease Control Rate (DCR) and Objective Response Rate (ORR) between immunotherapy and chemotherapy. The treatments are as follows: PembroplusCT = pembrolizumab + CT- SugeplusCT = sugemalimab + CT- CamreplusCT = camrelizumab + CT- AtezoplusCT = Atezolizumab + CT- TisleplusCT = tislelizumab + CT- SintiplusCT = sintilimab + CT- ToripplusCT = toripalimab + CT- IpiplusCT = ipilimumab + CT- NivoplusipiplusCT = nivolumab + ipilimumab + CT- DurvaplustremeplusCT = durvalumab + tremelimumab + CT- DurvaplusCT = durvalumab + CT

A total of 12 trials have reported the disease control rate (DCR) for 7 different therapies, while 17 trials have reported the objective response rate (ORR) for 11 therapies. These findings, as shown in Fig. 6A and B, Table S1, and Table S2, demonstrate that pembrolizumab has demonstrated a significant advantage in both DCR and ORR with respective values of DCR: 1.2 [1.1, 1.3] and ORR: 1.9 [1.5, 2.4]. However, no statistically significant differences have been observed between pembrolizumab and camrelizumab (DCR: 1.09 [0.93, 1.27], ORR: 1.08 [0.76, 1.55]), as well as tislelizumab (DCR: 1.06 [0.91, 1.22], ORR: 1.24 [0.9, 1.72]), in the comparison tables for both DCR and ORR.

#### 3.4.3 Safety

The safety analysis has revealed that treatment-related adverse events of Grade ≥3 were mainly associated with hematologic toxicities, such as anemia, neutropenia, thrombocytopenia, and leukopenia (refer to Table S3). In addition, there were other non-hematologic side effects observed, including fatigue, nausea, and pneumonia, but with a low incidence. The adverse events linked to PD-1 inhibitors were primarily fatigue, nausea, neutropenia, thrombocytopenia, and leukopenia. On the other hand, PD-L1-associated adverse events were mainly anemia and neutropenia. Among the different treatment options, pembrolizumab plus chemotherapy showed the highest incidences of anemia (34 %), fatigue (68 %), and nausea (59 %). The highest incidence of neutropenia (55.5 %) was associated with toripalimab plus chemotherapy. Sintilimab plus chemotherapy exhibited the highest incidences of thrombocytopenia (45.3 %) and leukopenia (36.3 %).

### 3.5 Results of subgroup analysis

The subgroup analysis was performed by the researchers, taking into account various factors such as age, gender, smoking status, pathology type, PD-L1 expression, organ metastasis, the administration of anti-PD-L1/PD-1 medications, and chemotherapeutic agents. The analysis focused on the primary endpoints of overall survival (OS) and progression-free survival (PFS), as indicated in Table S4.

#### 3.5.1 Metastasis of organs

Multiple studies have presented survival data concerning patients who have developed organ metastases, including liver metastases or brain metastases. To compare outcomes in patients with these organ metastases, the data were integrated, and subgroup analyses were conducted based on whether the patients had liver metastases or brain metastases.

##### 3.5.1.1 Hepatic metastases

**Fig. 7:**
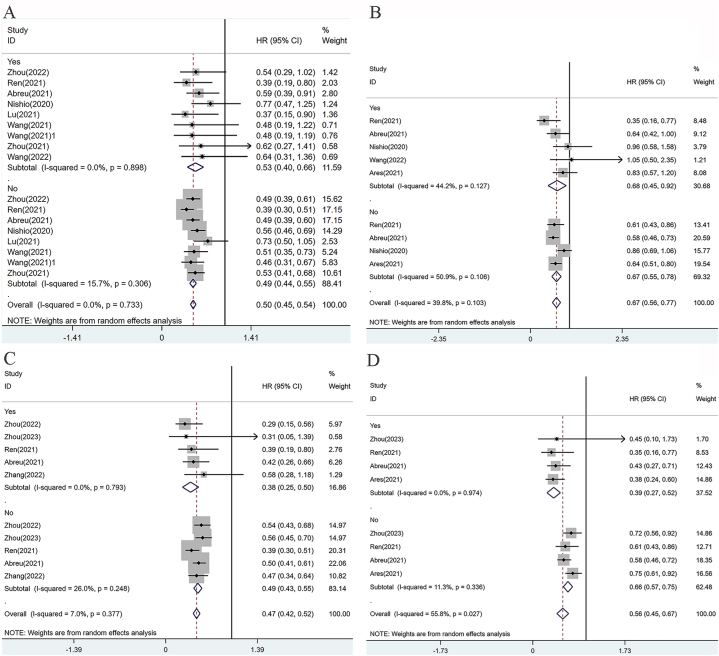
Subgroup analysis of OS and PFS in patients with different states of organ metastasis. **(A)** PFS for Liver Metastasis; **(B)** OS for Liver Metastasis; **(C)** PFS for Brain Metastasis; **(D)** OS for Brain Metastasis.

The results indicate that patients who underwent chemoimmunotherapy without liver metastases (0.49, [0.44, 0.55]) tended to have a longer progression-free survival (PFS) compared to those with liver metastases (0.53, [0.40, 0.66]) (Fig. 7A). The addition of sugemalimab (0.49, [0.39, 0.61]), camrelizumab (0.39, [0.30, 0.51]), pembrolizumab (0.49, [0.39, 0.60]), atezolizumab (0.56, [0.46, 0.69]), tislelizumab (0.51, [0.35, 0.73]), or sintilimab (0.53, [0.41, 0.68]) to chemotherapy significantly improved PFS compared to chemotherapy alone. Moreover, among patients with liver metastases, camrelizumab (0.39, [0.19, 0.80]), tislelizumab (0.37, [0.15, 0.90]), and pembrolizumab (0.59, [0.39, 0.91]) significantly enhanced their PFS, while sugemalimab (0.54, [0.29, 1.02]), atezolizumab (0.77, [0.47, 1.25]), sintilimab (0.62, [0.27, 1.41]), and toripalimab (0.64, [0.31, 1.36]) did not show statistically significant differences compared to chemotherapy alone. Regarding overall survival (OS) improvement (Fig. 7B), patients without liver metastases (0.67, [0.55, 0.78]) showed better responses to chemoimmunotherapies compared to those with liver metastases (0.68, [0.45, 0.92]). Camrelizumab (0.61, [0.43, 0.86]), pembrolizumab (0.58, [0.46, 0.73]), and nivolumab + ipilimumab (0.64, [0.51, 0.80]) in combination with chemotherapy were significantly more effective than chemotherapy alone in improving OS. However, there was no statistically significant difference observed between atezolizumab (0.86, [0.69, 1.06]) in combination with chemotherapy and chemotherapy alone. Among patients with liver metastases, camrelizumab (0.35, [0.16, 0.77]) was significantly more effective than chemotherapy alone, while no statistically significant difference was observed between chemotherapy alone and pembrolizumab (0.64, [0.42, 1.00]), atezolizumab (0.96, [0.58, 1.58]), toripalimab (1.05, [0.50, 2.35]), and nivolumab + ipilimumab (0.83, [0.57, 1.20]) in combination with chemotherapy.

##### 3.5.1.2 Brain metastases

Patients who have developed brain metastases showed longer progression-free survival (PFS) and overall survival (OS) compared to those without brain metastases. Specifically, in terms of PFS, the combination of sugemalimab plus chemotherapy (0.29, [0.15, 0.56]), camrelizumab plus chemotherapy (0.39, [0.19, 0.80]), and pembrolizumab plus chemotherapy (0.42, [0.26, 0.66]) demonstrated significantly greater efficacy when compared to chemotherapy alone (Fig. 7C). However, for patients with brain metastases from nonsquamous non-small cell lung cancer (NSCLC), the difference in efficacy between chemotherapy alone and camrelizumab plus chemotherapy (0.31, [0.05, 1.39]) as well as sintilimab plus chemotherapy (0.58, [0.28, 1.18]) was not statistically significant. On the other hand, in patients without brain metastases, the combinations of sugemalimab plus chemotherapy (0.54, [0.43, 0.68]), camrelizumab plus chemotherapy (0.56, [0.45, 0.70]), pembrolizumab plus chemotherapy (0.50, [0.41, 0.61]), and sintilimab plus chemotherapy (0.47, [0.34, 0.64]) significantly prolonged their PFS. The pooled analysis of OS in patients with brain metastases revealed that camrelizumab plus chemotherapy (0.35, [0.16, 0.77]), pembrolizumab plus chemotherapy (0.43, [0.27, 0.71]), and nivolumab + ipilimumab + chemotherapy (0.38, [0.24, 0.60]) exhibited higher efficacy compared to chemotherapy alone (Fig. 7D). In patients with non-squamous NSCLC and brain metastases, the difference in efficacy between camrelizumab plus chemotherapy and chemotherapy alone was not significant (0.45, [0.10, 1.73]). However, for patients without brain metastases, camrelizumab plus chemotherapy demonstrated better efficacy than chemotherapy alone in both squamous (0.61, [0.43, 0.86]) and non-squamous (0.72, [0.56, 0.92]) cancer patients.

#### 3.5.2 Chemotherapy drug

**Fig. 8:**
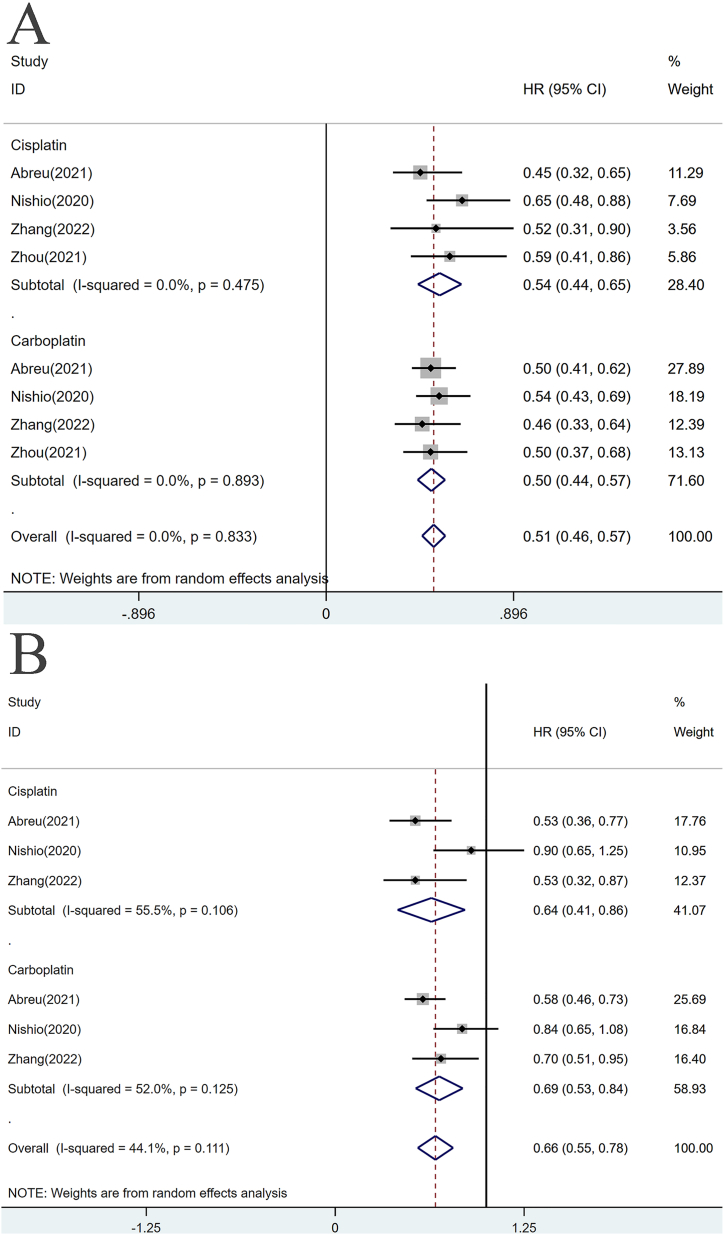
Subgroup analysis of OS and PFS in patients treated with different Chemotherapy drug. **(A)** PFS for Cisplatin/Carboplatin; **(B)** OS for Cisplatin/Carboplatin.

Several studies have observed differences in efficacy between different chemotherapeutic agents, specifically carboplatin and cisplatin. A combined analysis of progression-free survival (PFS) data revealed that carboplatin-based chemoimmunotherapies demonstrated higher efficacy compared to cisplatin-based chemoimmunotherapies when compared to chemotherapy alone, as shown in Fig. 8A. Furthermore, the combination of pembrolizumab, atezolizumab, and sintilimab with carboplatin-based regimens significantly improved PFS, with the most pronounced efficacy observed in the sintilimab group. Among the cisplatin-based regimens, pembrolizumab exhibited the most noteworthy efficacy in enhancing PFS compared to chemotherapy alone. Conversely, the analysis of overall survival (OS) data presented an opposite trend to that of PFS, as illustrated in Fig. 8B. Cisplatin-based chemoimmunotherapies displayed greater efficacy than carboplatin-based chemoimmunotherapies. However, no statistically significant difference in efficacy was observed between the atezolizumab group and the chemotherapy-alone group in either the cisplatin-based therapy or the carboplatin-based therapy. Pembrolizumab and sintilimab significantly extended the OS of patients compared with chemotherapy alone, regardless of the platinum-based therapy used.

#### 3.5.3 PD-L1 expression status, anti-PD-L1/PD-1 agents

PD-L1 TPS can be classified into four categories: PD-L1 TPS 1–49 %, PD-L1 TPS ≥50 %, PD-L1 TPS ≥1 %, and PD-L1 TPS <1 %. A total of 13 studies reported the outcomes of patients with PD-L1 TPS 1–49 % and PD-L1 TPS ≥50 %, while 15 studies reported the outcomes of patients with PD-L1 TPS ≥1 % and PD-L1 TPS <1 % (Fig. S1). There was no significant heterogeneity (I^2^ = 23.8 %, *p* = 00.080), and the fixed-effects model was applied. The findings revealed that immunotherapy, compared to chemotherapy, was found to offer advantages in extending overall survival (OS) for patients with advanced NSCLC, regardless of PD-L1 expression level (1–49 %: 0.75 [0.63, 0.87], ≥50 %: 0.65 [0.54, 0.76], ≥1 %: 0.67 [0.60, 0.74], <1 %: 0.70 [0.61, 0.79]). Additionally, immunotherapy showed benefits in terms of progression-free survival (PFS) for patients in all PD-L1 expression subgroups (1–49 %: 0.52 [0.43, 0.62], ≥50 %: 0.39 [0.33, 0.46], ≥1 %: 0.44 [0.39, 0.49], <1 %: 0.56 [0.49, 0.62]). Among these subgroups, patients with PD-L1 TPS ≥50 % showed the most significant clinical benefit in terms of both PFS and OS. However, even in patients with PD-L1 TPS <1 %, chemoimmunotherapy was observed to be superior to chemotherapy alone in terms of effectiveness.

A subgroup analysis was performed to compare the relative effectiveness of PD-1 and PD-L1 immune checkpoint inhibitors, based on the different types of immune checkpoints (Fig. S2). More studies utilized anti-PD-1 agents compared to anti-PD-L1 agents, and no significant variation was observed (I^2^ = 48.7 %, *p* = 0.018). Thus, we applied the fixed-effects model. The analysis indicated that combinations of anti-PD-1 drugs exhibited more significant survival benefits in terms of progression-free survival (PFS: 0.49 [0.45, 0.53]) and overall survival (OS: 0.62 [0.57, 0.68]) compared to combinations of anti-PD-L1 drugs (PFS: 0.59 [0.45, 0.72]; OS: 0.79 [0.66, 0.93]).

#### 3.5.4 Age and gender

In order to assess the impact of patient age on the effectiveness of ICIs, a threshold of 65 years was utilized. This threshold was employed to compare the efficacy between younger and middle-aged patients, and older patients. Subgroup analysis based on age (Fig. S3) revealed that patients under the age of 65 (0.48, [0.42, 0.53]) derived significantly greater benefit from combination ICI therapies compared to those aged 65 or above (0.55, [0.49, 0.62]) in terms of progression-free survival (PFS). Similar patterns were observed in the subgroup analysis for overall survival (OS), with patients under 65 years of age exhibiting a value of 0.68 [0.58, 0.77], and those aged 65 or above showing a value of 0.76 [0.68, 0.83]. Furthermore, subgroup analysis by gender was conducted (Fig. S4), and the results indicated varying responses to ICIs based on gender. It was found that there was a significant heterogeneity in gender across studies (I^2^ = 56.8 %, *p* = 0.001), thus the random-effects model was applied. The pooled analysis demonstrated that ICIs were more effective in improving PFS in females (0.49, [0.41, 0.56]) compared to males (0.50, [0.45, 0.56]). Conversely, the efficacy in improving OS was found to be greater in males (0.72, [0.65, 0.80]) than in females (0.74, [0.56, 0.92]).

#### 3.5.5 Smoking status and pathological type

According to the patients' smoking history, they were categorized into two groups: never smokers and previous or current smokers. The data were collected and subjected to meta-analysis (Fig. S5). The findings suggested that the chemoimmunotherapy treatment regimen significantly improved the progression-free survival (PFS) in patients who had previously or currently smoked (0.49, [0.44, 0.54]), compared to non-smokers (0.51, [0.40, 0.62]) receiving chemotherapy. Similar trends were observed for overall survival (OS) (previous or current smokers: 0.68, [0.59, 0.76]; non-smokers: 0.81, [0.58, 1.04]). Among the studies included in the analysis, seven studies enrolled patients with non-squamous NSCLC, while five studies enrolled patients with squamous NSCLC. All of these studies reported survival data. A pooled analysis of the data from these patients was conducted (Fig. S6), revealing that chemoimmunotherapies were more effective in prolonging PFS in patients with squamous carcinoma (0.51, [0.39, 0.62]) compared to those with non-squamous carcinoma (0.53, [0.48, 0.58]). However, the efficacy in extending OS was lower in squamous carcinoma patients compared to non-squamous patients (squamous: 0.68, [0.55, 0.81]; non-squamous: 0.65, [0.55, 0.74]).

### 3.6 Convergence, inconsistency, publication bias, and heterogeneity analysis

The included trials were depicted in the funnel plots, showing nearly symmetrical distributions, which indicated the absence of any apparent publication bias (Fig. S7). The analysis of OS heterogeneity revealed a ratio of 94.5 % of ipilimumab + CT to CT, while the analysis of PFS heterogeneity showed a ratio of 82.0 % of CT to camrelizumab + CT. The observed high heterogeneity may be attributed to the inclusion of patients with different pathological types of NSCLC (Fig. S8). The potential scaling factor was constrained to 1, as confirmed by the combined trace plot and density plot (Fig. S9), indicating the favorable convergence of this study (Fig. S10).

## 4 Discussion

The primary objective of this study was to evaluate and compare the effectiveness of immune checkpoint inhibitors (ICIs) in patients with advanced non-small cell lung cancer (NSCLC). The analysis was conducted based on data from 17 high-quality randomized controlled trials, encompassing over 7000 driver-gene-negative patients. The results of our analysis revealed that camrelizumab demonstrated the highest efficacy in terms of improving patients' progression-free survival (PFS). On the other hand, tislelizumab exhibited the most significant efficacy in enhancing patients' overall survival (OS). Moreover, pembrolizumab notably improved the objective response rate (ORR) and disease control rate (DCR) in patients. When analyzing the subgroups for OS, it was observed that patients with a programmed death-ligand 1 tumor proportion score (PD-L1 TPS) of 50 % or higher, those treated with anti-PD-1 drugs, patients below 65 years of age, male patients, smokers, those with non-squamous histology, patients without liver metastases, patients with brain metastases, and those receiving cisplatin-based therapy exhibited better efficacy compared to other subgroups. Conversely, female patients, patients with squamous carcinoma, and those on carboplatin-based regimens showed longer progression-free survival (PFS) in comparison. Overall, these findings provide valuable insights into the comparative effectiveness of different ICIs in the treatment of advanced NSCLC, highlighting specific subgroups that may benefit the most from each therapy.

In the treatment of advanced NSCLC [43], ICIs play a crucial role. The combination of Camrelizumab and chemotherapy in the CameL-sq trial has shown significant positive results for patients with non-small cell lung cancer (NSCLC). This combination therapy has demonstrated a significant improvement in both progression-free survival (PFS) and overall survival (OS), reducing the risk of death by 45 % [22]. Additionally, the Phase III RATIONALE-307 trial has provided further evidence for the potential benefits of immunotherapy in NSCLC treatment. This trial evaluated the addition of Tislelizumab to chemotherapy for patients with locally advanced or metastatic squamous NSCLC, irrespective of PD-L1 expression. The results showed a significant improvement in PFS, with an overall response rate (ORR) exceeding 70 % and a lower incidence of adverse events. These promising findings suggest that both Tislelizumab and Camrelizumab have the potential to be considered as optimal first-line treatment options for NSCLC [40]. Comparable outcomes were observed in various types of solid tumors. To assess the effectiveness and safety of tislelizumab in combination with chemotherapy as the initial treatment for patients with unresectable, locally advanced recurrent, or metastatic esophageal squamous cell carcinoma, a randomized, placebo-controlled, double-blind phase III study called RATIONALE-306 was conducted. The results showed a significant improvement in overall survival (OS) with the combination of tislelizumab and chemotherapy. In the entire patient population, the median OS was 17.2 months, which was longer than the 10.6 months observed in the placebo + chemotherapy group. Patients receiving tislelizumab and chemotherapy had a 34 % lower risk of death compared to those in the placebo + chemotherapy group (HR = 0.66, 95 % CI: [0.54, 0.80], *p* < 0.0001) [44]. In a single-arm phase II clinical trial, the efficacy of camrelizumab in combination with chemotherapy as neoadjuvant therapy for locally advanced head and neck squamous cell carcinoma was evaluated. The trial reported an objective response rate (ORR) of 96.7 % (29/30), and the disease-free survival rate at 12 months was 95.8 % (95 % CI: [73.9 %, 99.4 %]). The combination therapy also showed an acceptable safety profile [45]. However, given the small sample size of this trial, further clinical trials are necessary to explore the optimal efficacy of camrelizumab immunotherapy.

The variation in synergistic interactions between chemotherapy and immunotherapy is one reason for the difference in efficacy among different immunotherapy combinations. Several studies have shown that chemotherapy drugs not only have direct cytotoxic effects but also have an immunomodulatory role. They can induce immunogenic cell death or disrupt immunosuppressive tumor microenvironments [46]. This is especially important in non-immunogenic tumor microenvironments, where chemotherapy can potentially transform them into immunogenic ones. As a result, the combination of immunotherapy with chemotherapy has been observed to provide a survival advantage compared to chemotherapy alone [47].

In a recent meta-analysis conducted by Meng et al., which encompassed eight high-quality randomized controlled trials (RCTs), it was concluded that the treatment modality of immune checkpoint inhibitors (ICIs) combined with chemotherapy significantly enhanced overall survival (OS) (HR = 0.74, 95 % CI: [0.62, 0.85], *P* < 0.001) and progression-free survival (PFS) (HR = 0.66, 95 % CI: [0.57, 0.75], *P* < 0.001) for patients with advanced non-small cell lung cancer (NSCLC) when compared to chemotherapy alone. Notably, this treatment approach also displayed significant improvements in the survival rate for all patients, irrespective of their programmed death-ligand 1 (PD-L1) expression level [48]. Our study yielded similar findings, corroborating the efficacy of ICIs combined with chemotherapy in treating advanced NSCLC. Another recent meta-analysis, conducted by Lu et al., further explored the comparative effectiveness of different ICI monotherapies and combination therapies. Lu et al. performed a meta-analysis that incorporated 14 randomized controlled clinical trials, involving a total of 7823 patients with metastatic NSCLC. The results demonstrated that in cases of PD-L1-nonselective NSCLC, the group receiving nivolumab combined with ipilimumab exhibited improved progression-free survival (PFS) and objective response rate (ORR), while pembrolizumab notably prolonged patients' overall survival (OS). With regard to adverse events (AEs), nivolumab exhibited the lowest incidence rate [49]. These findings differ from those of our study, possibly due to the utilization of updated data from certain studies. For instance, our study incorporated extended follow-up survival data reported in 2023 from the Camel trial, as well as 5-year updated data reported in 2023 from the KEYNOTE-407 trial, in addition to data from the CHOICE-01 study. Another possible explanation for the discrepancy could be the selective inclusion of immunologic combination regimens (ICIs + chemotherapy, ICIs + ICIs + chemotherapy) in our study, excluding a comparison of the efficacy of immunologic monotherapy versus chemotherapy. This may have resulted in a reduced level of heterogeneity when compared to other studies. Furthermore, our study thoroughly incorporated all randomized controlled trials that met the inclusion criteria, making the comparative data more comprehensive and contributing to the divergence between our findings and those of other studies.

Our findings suggest that patients who receive immune checkpoint inhibitors (ICIs) and have liver metastases have a significantly worse prognosis compared to those without liver metastases, consistent with previous research. Liver metastases are associated with lower remission rate, PFS rate, and OS rate [[50], [51], [52]]. Lee et al. conducted a study to investigate the impact of intrahepatic tumors on antitumor immunity at distant SQ loci using a preclinical model. The results of the study showed that the presence of intrahepatic tumors leads to a significant reduction in systemic tumor-specific immunity in a PD-1-independent manner. This reduction is mediated by a liver-specific regulatory process, which causes significant changes in effector CD4^+^ and CD8^+^ T cells at distant tumor sites [53]. Around 20 % of non-small cell lung cancer (NSCLC) patients are diagnosed with brain metastases (BMs) at the time of diagnosis [54]. Moreover, approximately 25 %–50 % of patients develop brain metastases during the course of the disease [55,56]. Recent studies have shown the effectiveness and safety of immunotherapy in NSCLC patients with brain metastases [57,58]. Data from the CheckMate063, CheckMate 017, and CheckMate 057 trials demonstrated that patients with brain metastases from treated NSCLC had a longer median OS when treated with nivolumab compared to docetaxel [[59], [60], [61]]. In the OAK study, 85 patients with intracranial metastases, accounting for 10 % of the total population, were enrolled. Among these patients, 38 received treatment with the PD-L1 inhibitor atezolizumab, which resulted in a prolonged median survival of approximately 8.2 months (20.1 vs. 11.9 months). Additionally, patients in the test group who did not have brain metastases at the time of enrollment had a significantly longer time before developing brain metastases compared to the chemotherapy control group [62]. In the context of lung cancer brain metastases, animal models have demonstrated that patients with extracranial metastases show a higher response rate to ICI compared to those without extracranial metastases. Further studies have shown that the increased tumor response is not due to an increased number of craniofacial infiltrating lymphocytes. Instead, extracranial T cells are transported to the intracranium to exert their killing effect, involving the mobilization of a limited number of tumor-infiltrating lymphocytes (TILs) and the concentration of T cells for killing brain metastases [[63], [64], [65], [66]]. Combining immunotherapy with conventional lung cancer treatment modalities has shown an overall effectiveness rate of 10 %–56 % for brain metastases, providing a new treatment option for NSCLC patients with brain metastases [67].

Finding the most suitable partner for immune checkpoint inhibitors (ICIs) is an urgent priority. Specifically, it is crucial to identify the optimal chemotherapy drugs to be combined with ICIs for achieving the best outcomes in patients with non-small cell lung cancer (NSCLC). However, there is currently a lack of direct comparative studies in this area. Several clinical trials, namely KEYNOTE-189, Impower-132, and ORIENT-11, have reported survival data for patients receiving carboplatin and cisplatin, respectively. In our network meta-analysis, we have found that carboplatin-based chemoimmunotherapies significantly extend progression-free survival (PFS) of patients (0.50, [0.44, 0.57]), while cisplatin-based chemoimmunotherapies demonstrate advantages in improving overall survival (OS) (0.64, [0.41, 0.86]). Cisplatin, a first-generation platinum-based antitumor drug, has shown effectiveness in treating various cancers including testicular cancer, lung cancer, ovarian cancer, and head and neck tumors [68]. On the other hand, carboplatin is a derivative of cisplatin that exhibits higher water solubility due to the replacement of two chloride ions on the cisplatin molecule with a cyclobutane-dicarboxylic acid, showing a 16-fold increase in solubility [69]. Platinum-based anticancer agents, such as carboplatin and cisplatin, target DNA by forming complexes with guanine or thymine, and their electrophilic nature allows them to react with nucleophilic residues in DNA molecules, leading to tumor cell necrosis or apoptosis [[70], [71], [72]]. The POSEIDON study provided overall survival data for other chemotherapeutic agents. It showed no statistically significant differences in OS between nab-paclitaxel-based, pemetrexed-based, and gemcitabine-based single-agent immune-combination regimens when compared with chemotherapy-alone regimens. However, in the dual-immunotherapy group, the combination regimen containing pemetrexed demonstrated superior efficacy compared to those containing nab-paclitaxel and gemcitabine [73]. Currently, gemcitabine, pemetrexed, nab-paclitaxel, and platinum-based cytotoxic chemotherapeutic agents are considered standard treatments for NSCLC patients [74], but further head-to-head comparison data are needed to evaluate the optimal chemotherapeutic agents to combine with ICIs. Therefore, our findings need validation through extensive future clinical studies.

The study's strength lies in its comprehensive and meticulous analysis of the currently available trial data. It not only compared the effectiveness of first-line immune checkpoint inhibitors (ICIs) combined with chemotherapy in driver-gene negative advanced non-small cell lung cancer (NSCLC), but also conducted subgroup analyses based on patient characteristics such as age, gender, smoking status, pathology type, PD-L1 expression, organ metastasis, and specific drugs used. This approach allows clinicians to tailor the treatment regimen to the individual patients' specific characteristics. The subgroup analyses revealed varying therapeutic effects based on different levels of PD-L1 tumor proportion score (TPS). In recent years, the use of PD-L1 expression as a prognostic and predictive indicator for treatment has gained biological rationale, given the mechanism of action of ICIs [75,76]. However, there is still controversy surrounding this issue [77,78]. Shen et al. conducted a meta-analysis on PD-L1 expression and the efficacy of ICIs across different types of tumors, and their findings suggested a preference for PD-L1 or PD-L1 blocking therapy over traditional therapy for patients with negative PD-L1 expression. Therefore, relying solely on PD-L1 expression is not enough to determine which patients should receive PD-1 or PD-L1 blocking therapy [79].

The effectiveness of ICI monotherapy can be predicted by specific biomarkers, including TMB and dMMR/MSI, as shown by some studies [80]. Additionally, recent research has highlighted the importance of ctDNA in various aspects of cancer management. In breast cancer, ctDNA can predict recurrence, evaluate the response to neoadjuvant therapy, and determine prognosis. In colon cancer, it can guide adjuvant therapy, while in lung cancer, it can assist in anti-EGFR therapy. Moreover, ctDNA can provide insights into the mechanisms of drug resistance in rectal cancer [[81], [82], [83], [84], [85], [86]]. However, the routine clinical use of ctDNA is presently limited due to technological constraints in detection and tumor staging.

A recent study conducted by Zhou et al. investigated the significance of HLA class I molecular differentiation differences (HED) in predicting and prognosticating the outcomes of NSCLC patients who received first-line PD-1 blockade in combination with chemotherapy. The study, which analyzed the clinical, genomic, and survival data of 427 patients from two phase III clinical trials (CameL and CameL-sq), found a correlation between high HED levels and improved objective response rate (ORR), progression-free survival (PFS), and overall survival (OS). Moreover, the study demonstrated that combining HED^high^ with PD-L1 positive expression resulted in better predictive performance for OS (0.29, [0.12, 0.69]; *P* = 0.003), ORR (84.2 % vs. 52.1 %; *P* = 0.025), and PFS (0.41, [0.22, 0.80]; *P* = 0.006) compared to the chemotherapy-alone group [87]. This study contributes valuable insights into the identification of biomarkers for combination therapy.

To control for confounding factors, we eliminated low-quality studies, such as the EMPOWER-Lung 3 study [88]. Although this study investigated the impact of cemiplimab combined with platinum-based chemotherapy as a primary treatment for advanced NSCLC, it included 58 patients who had previously received radiotherapy and/or systemic adjuvant therapy. Hence, we excluded this study from our analysis. Additionally, we performed sensitivity analyses and subgroup analyses to ensure the study findings' credibility.

This study is subject to several limitations. Firstly, there was inconsistency in the staging of the patients included in the 17 randomized controlled trials. Approximately 53 % of the studies enrolled patients with stage IIIb-IV, while 47 % enrolled patients with stage IV, resulting in population heterogeneity to a certain extent. Hence, a random effects model was utilized in the meta-analysis. Secondly, although the included studies were strictly randomized controlled trials, in some cases, randomization was stratified by PD-L1 expression, leading to an imbalance of patients between the two treatment groups. Thirdly, the variation in synergy between chemotherapy and immunotherapy also contributes to a certain degree of heterogeneity, underscoring the importance of identifying the most effective partner for immunotherapy. Fourthly, certain subgroup analyses had a limited number of trials, such as the organ metastasis subgroups and cisplatin/carboplatin subgroups. This limits the reliability of the obtained results and requires cautious interpretation. We intend to closely monitor relevant clinical trials and perform another pooled analysis targeting patients with organ metastasis and specific chemotherapeutic agents once sufficient data are available. Fifthly, our main conclusions rely on a relatively small number of clinical trials, and the comparisons between different treatment regimens are based on indirect comparisons. Therefore, additional trials are necessary to validate these findings. Sixthly, each trial had a different follow-up period, and some of the overall survival (OS) data from included trials were not sufficiently mature due to limited follow-up. Additionally, during the study screening process, certain trials (such as IMpower130 [89], IMpower131 [90] and CheckMate 227 [91]) did not meet the criteria for inclusion in our analysis as they focused on different aspects compared to other meta-analyses and contradicted the objective of our study.

The application of immunotherapy in the treatment of malignant tumors has reached a mature stage, and its effectiveness in treating non-small cell lung cancer (NSCLC) is gradually being established. However, the use of immune checkpoint inhibitors (ICIs) can activate T-cells that in turn may cause adverse events in different organs, including the endocrine glands, gastrointestinal tract, skin, lungs, liver, kidneys, neurological system, muscles, and blood [73,[92], [93], [94], [95]]. To address these challenges, it is crucial to develop a tailored chemoimmunotherapy regimen for patients, enhancing the efficacy of combination therapy while minimizing adverse effects. Furthermore, the accurate screening of patients who are suitable for combination therapy and the exclusion of those for whom it is contraindicated are essential. Currently, numerous clinical trials are underway to investigate the potential of combining immunotherapy with anti-vascular or targeted therapies. The results of these trials hold great promise for bringing significant advancements in cancer treatment.

## 5 Conclusion

Tislelizumab plus chemotherapy, camrelizumab plus chemotherapy, and pembrolizumab plus chemotherapy emerged as the most effective combinations among the various options of immune checkpoint inhibitors (ICIs) and ICIs combined with chemotherapy. These combinations demonstrated notable improvements in overall survival (OS), progression-free survival (PFS), disease control rate (DCR), and objective response rate (ORR) for patients. In terms of safety, the predominant hematologic toxicities observed were anemia, neutropenia, and leukopenia. Common non-hematologic toxicities included nausea and fatigue. Considering the limitations of this study, further research is necessary to gain a better understanding of the efficacy and safety profiles of these treatment combinations.

## Ethics approval and consent to participate

Not applicable.

## Data availability statement

Data included in article/supp. material/referenced in article.

## Funding

This work was supported by grants from the Science and Technology Agency of Qinghai Province (2022-ZJ-719).

## CRediT authorship contribution statement

**Xuewen Zhang:** Writing – original draft, Conceptualization. **Min Wu:** Methodology. **Jie Chen:** Formal analysis. **Kaiman Zheng:** Formal analysis. **Huchen Du:** Supervision. **Bo Li:** Resources. **Yujia Gu:** Supervision. **Jun Jiang:** Writing – review & editing, Funding acquisition.

## Declaration of competing interest

The authors declare that they have no known competing financial interests or personal relationships that could have appeared to influence the work reported in this paper.
