# Supplementary material for: Comparative efficacy of immune checkpoint inhibitors combined with chemotherapy in patients with advanced driver-gene negative non-small cell lung cancer: A systematic review and network meta-analysis
Source: Heliyon. 2024 May 7;10(10):e30809. doi: 10.1016/j.heliyon.2024.e30809 (PMC11107224; doi:10.1016/j.heliyon.2024.e30809)
Supplement: Multimedia component 1 [file mmc1.docx]

**Supplementary material 1:** Search strategy

Pubmed

| (("Carcinoma, Non-Small-Cell Lung"[Mesh]) OR (((((((((((((Carcinoma, Non-Small-Cell Lung[Title/Abstract]) OR (Carcinoma, Non Small Cell Lung[Title/Abstract])) OR (Carcinomas, Non-Small-Cell Lung[Title/Abstract])) OR (Lung Carcinoma, Non-Small-Cell[Title/Abstract])) OR (Lung Carcinomas, Non-Small-Cell[Title/Abstract])) OR (Non-Small-Cell Lung Carcinomas[Title/Abstract])) OR (Non-Small-Cell Lung Carcinoma[Title/Abstract])) OR (Non Small Cell Lung Carcinoma[Title/Abstract])) OR (Carcinoma, Non-Small Cell Lung[Title/Abstract])) OR (Non-Small Cell Lung Carcinoma[Title/Abstract])) OR (Non-Small Cell Lung Cancer[Title/Abstract])) OR (Nonsmall Cell Lung Cancer[Title/Abstract])) OR (NSCLC[Title/Abstract]))) AND (("Immune Checkpoint Inhibitors"[Mesh]) OR ((((((((((((((((((((((((((((((((((Immune checkpoint inhibitors[Title/Abstract]) OR (Checkpoint Inhibitors, Immune[Title/Abstract])) OR (Immune Checkpoint Inhibitor[Title/Abstract])) OR (Checkpoint Inhibitor, Immune[Title/Abstract])) OR (Immune Checkpoint Blockers[Title/Abstract])) OR (Checkpoint Blockers, Immune[Title/Abstract])) OR (Immune Checkpoint Blockade[Title/Abstract])) OR (Checkpoint Blockade, Immune[Title/Abstract])) OR (Immune Checkpoint Inhibition[Title/Abstract])) OR (Checkpoint Inhibition, Immune[Title/Abstract])) OR (PD-L1 Inhibitors[Title/Abstract])) OR (PD L1 Inhibitors[Title/Abstract])) OR (PD-L1 Inhibitor[Title/Abstract])) OR (PD L1 Inhibitor[Title/Abstract])) OR (Programmed Death-Ligand 1 Inhibitors[Title/Abstract])) OR (Programmed Death Ligand 1 Inhibitors[Title/Abstract])) OR (PD-1-PD-L1 Blockade[Title/Abstract])) OR (Blockade, PD-1-PD-L1[Title/Abstract])) OR (PD 1 PD L1 Blockade[Title/Abstract])) OR (CTLA-4 Inhibitors[Title/Abstract])) OR (CTLA 4 Inhibitors[Title/Abstract])) OR (CTLA-4 Inhibitor[Title/Abstract])) OR (CTLA 4 Inhibitor[Title/Abstract])) OR (Cytotoxic T-Lymphocyte-Associated Protein 4 Inhibitors[Title/Abstract])) OR (Cytotoxic T Lymphocyte Associated Protein 4 Inhibitors[Title/Abstract])) OR (Cytotoxic T-Lymphocyte-Associated Protein 4 Inhibitor[Title/Abstract])) OR (Cytotoxic T Lymphocyte Associated Protein 4 Inhibitor[Title/Abstract])) OR (PD-1 Inhibitors[Title/Abstract])) OR (PD 1 Inhibitors[Title/Abstract])) OR (PD-1 Inhibitor[Title/Abstract])) OR (Inhibitor, PD-1[Title/Abstract])) OR (PD 1 Inhibitor[Title/Abstract])) OR (Programmed Cell Death Protein 1 Inhibitor[Title/Abstract])) OR (Programmed Cell Death Protein 1 Inhibitors[Title/Abstract]))) |
| --- |

Embase

| No. | Query |
| --- | --- |
| #49 | #15 AND #48 |
| #48 | #16 OR #17 OR #18 OR #19 OR #20 OR #21 OR #22 OR #23 OR #24 OR #25 OR #26 OR  #27 OR #28 OR #29 OR #30 OR #31 OR #32 OR #33 OR #34 OR #35 OR #36 OR #37 OR  #38 OR #39 OR #40 OR #41 OR #42 OR #43 OR #44 OR #45 OR #46 OR #47 |
| #47 | 'programmed cell death protein 1 inhibitor':ab,ti |
| #46 | 'programmed cell death protein 1 inhibitors':ab,ti |
| #45 | 'inhibitor, pd-1':ab,ti |
| #44 | 'pd-1 inhibitor':ab,ti |
| #43 | 'pd-1 inhibitors':ab,ti |
| #42 | 'cytotoxic t lymphocyte associated protein 4 inhibitor':ab,ti |
| #41 | 'cytotoxic t lymphocyte associated protein 4 inhibitors':ab,ti |
| #40 | 'ctla 4 inhibitor':ab,ti |
| #39 | 'ctla-4 inhibitor':ab,ti |
| #38 | 'ctla 4 inhibitors':ab,ti |
| #37 | 'ctla-4 inhibitors':ab,ti |
| #36 | 'pd 1 pd l1 blockade':ab,ti |
| #35 | 'blockade, pd-1-pd-l1':ab,ti |
| #34 | 'pd-1-pd-l1 blockade':ab,ti |
| #33 | 'programmed death ligand 1 inhibitors':ab,ti |
| #32 | 'programmed death-ligand 1 inhibitors':ab,ti |
| #31 | 'pd l1 inhibitor':ab,ti |
| #30 | 'pd-l1 inhibitor':ab,ti |
| #29 | 'pd l1 inhibitors':ab,ti |
| #28 | 'pd-l1 inhibitors':ab,ti |
| #27 | 'checkpoint inhibition, immune':ab,ti |
| #26 | 'immune checkpoint inhibition':ab,ti |
| #25 | 'checkpoint blockade, immune':ab,ti |
| #24 | 'checkpoint blockade, immune':ab,ti |
| #23 | 'immune checkpoint blockade':ab,ti |
| #22 | 'checkpoint blockers, immune':ab,ti |
| #21 | 'immune checkpoint blockers':ab,ti |
| #20 | 'checkpoint inhibitor, immune':ab,ti |
| #19 | 'immune checkpoint inhibitor':ab,ti |
| #18 | 'checkpoint inhibitors, immune':ab,ti |
| #17 | 'immune checkpoint inhibitor':ab,ti |
| #16 | 'immune checkpoint inhibitor'/exp |
| #15 | #1 OR #2 OR #3 OR #4 OR #5 OR #6 OR #7 OR #8 OR #9 OR #10 OR #11 OR #12 OR  #13 OR #14 |
| #14 | 'nsclc':ab,ti |
| #13 | 'nonsmall cell lung cancer':ab,ti |
| #12 | 'non-small cell lung cancer':ab,ti |
| #11 | 'non-small cell lung carcinoma':ab,ti |
| #10 | 'carcinoma, non-small cell lung':ab,ti |
| #9 | 'non small cell lung carcinoma':ab,ti |
| #8 | 'non-small-cell lung carcinoma':ab,ti |
| #7 | 'non-small-cell lung carcinomas':ab,ti |
| #6 | 'lung carcinomas, non-small-cell':ab,ti |
| #5 | 'lung carcinoma, non-small-cell':ab,ti |
| #4 | 'carcinomas, non-small-cell lung':ab,ti |
| #3 | 'carcinoma, non small cell lung':ab,ti |
| #2 | 'non small cell lung cancer':ab,ti |
| #1 | 'non small cell lung cancer'/exp |

Web of Science

| TS=(Carcinoma, Non-Small-Cell Lung) OR TS=(Carcinoma, Non Small Cell Lung) OR TS=(Carcinomas, Non-Small-Cell Lung) OR TS=(Lung Carcinoma, Non-Small-Cell) OR TS=(Lung Carcinomas, Non-Small-Cell) OR TS=(Non-Small-Cell Lung Carcinomas) OR TS=(Non-Small-Cell Lung Carcinoma) OR TS=(Non Small Cell Lung Carcinoma) OR TS=(Carcinoma, Non-Small Cell Lung) OR TS=(Non-Small Cell Lung Carcinoma) OR TS=(Non-Small Cell Lung Cancer) OR TS=(Nonsmall Cell Lung Cancer) OR TS=(NSCLC)  TS=(Immune checkpoint inhibitors) OR TS=(Checkpoint Inhibitors, Immune) OR TS=(Immune Checkpoint Inhibitor) OR TS=(Checkpoint Inhibitor, Immune) OR TS=(Immune Checkpoint Blockers) OR TS=(Checkpoint Blockers, Immune) OR TS=(Immune Checkpoint Blockade) OR TS=(Checkpoint Blockade, Immune) OR TS=(Immune Checkpoint Inhibition) OR TS=(Checkpoint Inhibition, Immune) OR TS=(PD-L1 Inhibitors) OR TS=(PD L1 Inhibitors) OR TS=(PD-L1 Inhibitor) OR TS=(PD L1 Inhibitor) OR TS=(Programmed Death-Ligand 1 Inhibitors) OR TS=(Programmed Death Ligand 1 Inhibitors) OR TS=(PD-1-PD-L1 Blockade) OR TS=(Blockade, PD-1-PD-L1) OR TS=(PD 1 PD L1 Blockade) OR TS=(CTLA-4 Inhibitors) OR TS=(CTLA 4 Inhibitors) OR TS=(CTLA-4 Inhibitor) OR TS=(CTLA 4 Inhibitor) OR TS=(Cytotoxic T-Lymphocyte-Associated Protein 4 Inhibitors) OR TS=(Cytotoxic T Lymphocyte Associated Protein 4 Inhibitors) OR TS=(Cytotoxic T-Lymphocyte-Associated Protein 4 Inhibitor) OR TS=(Cytotoxic T Lymphocyte Associated Protein 4 Inhibitor) OR TS=(PD-1 Inhibitors) OR TS=(PD 1 Inhibitors) OR TS=(PD-1 Inhibitor) OR TS=(Inhibitor, PD-1) OR TS=(PD 1 Inhibitor) OR TS=(Programmed Cell Death Protein 1 Inhibitor) OR TS=(Programmed Cell Death Protein 1 Inhibitors)  TS=(Chemical treatment) OR TS=(Chemical treatment) OR TS=(Chemotherapy) OR TS=(Chemotherapies) OR TS=(chemo) OR TS=(chemotherapeutic)  TS=(Drive-negative) OR TS=(Gene negative) |
| --- |

Cochrane

| #1 | MeSH descriptor: [Carcinoma, Non-Small-Cell Lung] explode all trees |
| --- | --- |
| #2 | (Carcinoma, Non-Small-Cell Lung):ti, ab, kw OR (Carcinoma, Non Small Cell Lung):ti, ab, kw OR  (Carcinomas, Non-Small-Cell Lung):ti, ab, kw OR (Lung Carcinoma, Non-Small-Cell):ti,ab,kw OR  (Lung Carcinomas, Non-Small-Cell):ti,ab,kw |
| #3 | (Non-Small-Cell Lung Carcinomas):ti, ab,kw OR (Non-Small-Cell Lung Carcinoma):ti, ab,kw OR  (Non Small Cell Lung Carcinoma):ti, ab, kw OR (Carcinoma, Non-Small Cell Lung):ti,ab, kw OR  (Non-Small Cell Lung Carcinoma):ti,ab,kw |
| #4 | (Non-Small Cell Lung Cancer):ti, ab, kw OR (Nonsmall Cell Lung Cancer):ti,ab,kw OR (NSCLC):ti,ab, kw |
| #5 | #1 OR #2 OR #3 OR #4 |
| #6 | MeSH descriptor: [Immune Checkpoint Inhibitors] explode all trees |
| #7 | (Immune checkpoint inhibitors):ti, ab, kw OR (Checkpoint Inhibitors, Immune):ti, ab, kw OR  (Immune Checkpoint Inhibitor):ti,ab, kw OR (Checkpoint Inhibitor, Immune):ti,ab, kw OR  (Immune Checkpoint Blockers):ti, ab, kw |
| #8 | (Checkpoint Blockers, Immune):ti, ab, kw OR (Immune Checkpoint Blockade):ti, ab, kw OR  (Checkpoint Blockade, Immune):ti, ab, kw OR (Immune Checkpoint Inhibition):ti, ab, kw OR  (Checkpoint Inhibition, Immune):ti,ab, kw |
| #9 | (PD-L1 Inhibitors):ti, ab, kw OR (PD L1 Inhibitors):ti, ab,kw OR (PD-L1 Inhibitor):ti, ab,kw OR  (PD L1 Inhibitor):ti, ab, kw OR (Programmed Death-Ligand 1 |
| #10 | (Programmed Death Ligand 1 Inhibitors):ti, ab, kw |
| #11 | (CTLA 4 Inhibitors):ti, ab, kw OR (CTLA-4 Inhibitor):ti,ab,kw OR (CTLA 4 Inhibitor):ti,ab,kw OR  (Cytotoxic T-Lymphocyte-Associated Protein 4 Inhibitors):ti, ab, kw OR  (Cytotoxic T Lymphocyte Associated Protein 4 Inhibitors):ti, ab, kw |
| #12 | (Cytotoxic T-Lymphocyte-Associated Protein 4 Inhibitor):ti,ab,kw OR  (Cytotoxic T Lymphocyte Associated Protein 4 Inhibitor):ti, ab,kw OR (PD-1 Inhibitors):ti, ab,kw OR (PD 1 Inhibitors):ti, ab, kw OR (PD-1 Inhibitor):ti, ab,kw |
| #13 | (Inhibitor, PD-1):ti, ab,kw OR (PD 1 Inhibitor):ti, ab,kw OR (Programmed Cell Death Protein 1 Inhibitor):ti, ab,kw OR  (Programmed Cell Death Protein 1 Inhibitors):ti, ab,kw OR (ICI):ti, ab, kw |
| #14 | #6 OR #7 OR #8 OR #9 OR #10 OR #11 OR #12 OR #13 |
| #15 | #5 AND #14 |
