# Supplementary material for: Comparative efficacy of immune checkpoint inhibitors combined with chemotherapy in patients with advanced driver-gene negative non-small cell lung cancer: A systematic review and network meta-analysis
Source: Heliyon. 2024 May 7;10(10):e30809. doi: 10.1016/j.heliyon.2024.e30809 (PMC11107224; doi:10.1016/j.heliyon.2024.e30809)
Supplement: Multimedia component 2 [file mmc2.docx]

**Table S1** Characteristics of the included studies.

|  |  |  |  |  |  |  |  |  |  |  |  |  |  | Age, years | | Sex | | Smoking status | | Tumour pathological type | | Brain metastases | | Liver metastases | | \| Platinum chemotherapy \| \| --- \| | |
| --- | --- | --- | --- | --- | --- | --- | --- | --- | --- | --- | --- | --- | --- | --- | --- | --- | --- | --- | --- | --- | --- | --- | --- | --- | --- | --- | --- | --- |
| **study** | **treatment** | **PD-L1 high; ≥50% (%)** | **PFS** | **OS** | **PD-L1 low; ≥1–49% (%)** | **PFS** | **OS** | **PD-L1 positive; ≥1% (%)** | **PFS** | **OS** | **PD-L1 negative; <1% (%)** | **PFS** | **OS** | <65 | ≥65 | Male | Female | Never smoked | Current or former smoker | Non-squamous | Squamous | Yes | No | Yes | No | Cisplatin | Carboplatin |
| Awad (2021) | PembroplusCT | 20 (33) | NR | NR | 19 (32) | NR | NR | 39 (65) | 0.70 (0.40-1.22) | 0.84 (0.47-1.50) | 21 (35) | 0.35 (0.17-0.72) | 0.54 (0.26-1.13) | NR | NR | NR | NR | NR | NR | NR | NR | NR | NR | NR | NR | NR | NR |
|  | CT | 17 (27) |  |  | 23 (37) |  |  | 40 (63) |  |  | 23 (37) |  |  | NR | NR | NR | NR | NR | NR | NR | NR | NR | NR | NR | NR | NR | NR |
| Zhou (2022) | SugeplusCT | 104(30) | 0.41 (0.27-0.62) | NR | 92(29) | 0.53 (0.35-0.79) | NR | 196 (61) | 0.46 (0.35-0.62) | NR | 124 (39) | 0.56 (0.40-0.77) | NR | 0.48 (0.36-0.63) | 0.53 (0.38-0.75) | 0.48 (0.38-0.62) | 0.60 (0.37-0.99) | 0.55 (0.36-0.83) | 0.48 (0.37-0.62) | 0.59 (0.45-0.79) | 0.34 (0.24-0.48) | 0.29 (0.15-0.56) | 0.54 (0.43–0.68) | 0.54 (0.29-1.02) | 0.49 (0.39-0.61) | NR | NR |
|  | CT | 47(30) |  |  | 48(30) |  |  | 95 (60) |  |  | 64 (40) |  |  |  |  |  |  |  |  |  |  |  |  |  |  | NR | NR |
| Zhou (2023) | CamreplusCT | 30 (15) | 0.42 (0.19-0.87) | 0.68 (0.28-1.63) | 108 (53) | 0.59 (0.42-0.81) | 0.76 (0.53-1.08) | 138 (67) | 0.52 (0.39-0.69) | 0.70 (0.51-0.97) | 49 (24) | 0.75 (0.50-1.13) | 0.84 (0.55-1.27) | PFS:0.56 (0.43-0.72) | 0.53 (0.32-0.86) | 0.53 (0.41-0.68) | 0.62 (0.41-0.95) | 0.63 (0.44-0.90) | 0.50 (0.38-0.67) | NR | NR | PFS:0.31 (0.05-1.39) | PFS:0.56 (0.45-0.70) | NR | NR | NR | NR |
|  | CT | 20 (10) |  |  | 97 (47) |  |  | 117 (57) |  |  | 69 (33) |  |  | OS:0.74 (0.56-0.98) | 0.71 (0.43-1.15) | 0.78 (0.59-1.03) | 0.57 (0.35-0.92) | 0.69 (0.45-1.04) | 0.73 (0.54-0.98) | NR | NR | OS:0.45 (0.10-1.73) | OS:0.72 (0.56-0.92) | NR | NR | NR | NR |
| Ren (2021) | CamreplusCT | 37 (19) | 0.30(0.17-0.55) | 0.48 (0.21-1.12) | 58 (30) | 0.32(0.20-0.51) | 0.52 (0.27-1.00) | 95 (49) | 0.34(0.24-0.49) | 0.52 (0.31-0.86) | 91 (47) | 0.49(0.35-0.68) | 0.62 (0.41-0.94) | PFS:0.34(0.25-0.47) | 0.49(0.33-0.71) | 0.35 (0.27-0.46) | 0.97 (0.40-2.34) | 0.77 (0.38-1.54) | 0.34  (0.26-0.45) | NR | NR | 0.39 (0.19-0.80) | | 0.39 (0.30-0.51) | | NR | NR |
|  | CT | 44 (22) |  |  | 49 (25) |  |  | 93 (47) |  |  | 97 (49) |  |  | OS:0.51 (0.34-0.77) | 0.64 (0.40-1.04) | 0.55 (0.40-0.75) | 1.06 (0.21-5.26) | 0.92 (0.31-2.73) | 0.52 (0.37-0.73) | NR | NR | 0.35 (0.16-0.77) | | 0.61 (0.43-0.86) | | NR | NR |
| Abreu (2021) | PembroplusCT | 132 (32) | NR | NR | 128 (31) | NR | NR | 260 (63) | 0.42 (0.33-0.53) | 0.63 (0.48‒0.81) | 127 (31) | 0.67 (0.49–0.93) | 0.51 (0.36-0.71) | PFS:0.42 (0.32-0.55) | 0.63 (0.48-0.83) | 0.58 (0.46–0.74) | 0.39 (0.29-0.52) | 0.46 (0.26-0.79) | 0.50 (0.41-0.60) | NR | NR | 0.42 (0.26-0.66) | 0.50 (0.41-0.61) | 0.59 (0.39-0.91) | 0.49 (0.39-0.60) | 0.45  (0.32–0.65) | 0.50 (0.41–0.62) |
|  | CT | 70 (34) |  | NR | 58 (28) | NR | NR | 128 (62) |  |  | 63 (31) |  |  | OS:0.49 (0.37-0.65) | 0.72 (0.54-0.97) | 0.74 (0.56–0.96) | 0.41 (0.30-0.56) | 0.30 (0.16-0.54) | 0.62 (0.50-0.77) | NR | NR | 0.43 (0.27-0.71) | 0.58 (0.46‒0.72) | 0.64 (0.42-1.00) | 0.58 (0.46-0.73) | 0.53  (0.36–0.77) | 0.58 (0.46–0.73) |
| Nishio (2020) | AtezoplusCT | 25 (14.2) | 0.46 (0.22-0.96) | 0.73 (0.31-1.73) | 63 (35.8) | 0.80 (0.56-1.16) | 1.18 (0.80-1.76) | 88(50) | NR | NR | 88 (50.0) | 0.45 (0.31-0.64) | 0.67 (0.46-0.96) | PFS:0.63 (0.49-0.80) | 0.55 (0.42-0.73) | 0.64 (0.51-0.79) | 0.51 (0.36-0.71) | 0.49 (0.28-0.87) | 0.61 (0.50-0.74) | NR | NR | NR | NR | 0.77 (0.47-1.25) | 0.56 (0.46-0.69) | 0.65 (0.48, 0.88) | 0.54 (0.43, 0.69) |
|  | CT | 20 (11.9) |  |  | 73 (43.5) |  |  | 93(55.4) | NR | NR | 75 (44.6) |  |  | OS:0.88 (0.67-1.16) | 0.84 (0.63-1.13) | 0.93 (0.73-1.18) | 0.76 (0.54-1.09) | 0.78 (0.42-1.43) | 0.89 (0.72-1.09) | NR | NR | NR | NR | 0.96 (0.58-1.58) | 0.86 (0.69-1.06) | 0.90 (0.65, 1.25) | 0.84 (0.65, 1.08) |
| Novello (2023) | PembroplusCT | 73 (26.3) | 0.48(0.33-0.69) | 0.68  (0.47-0.97) | 103 (37.1) | 0.60(0.45-0.81) | 0.61(0.45-0.83) | 176 (63.3) | NR | NR | 95 (34.2) | 0.70(0.52-0.95) | 0.83(0.61-1.13) | NR | NR | NR | NR | NR | NR | NR | NR | NR | NR | NR | NR | NR | NR |
|  | CT | 73 (26.0) |  |  | 104 (37.0) |  |  | 177 (63.0) | NR | NR | 99 (35.2) |  |  | NR | NR | NR | NR | NR | NR | NR | NR | NR | NR | NR | NR | NR | NR |
| Lu (2021) | TisleplusCT | 74 (33.2) | 0.308 (0.167-0.567) | NR | 53 (23.8) | 1.058 (0.507-2.209) | NR | 127(57) | 0.549 (0.347-0.869) | NR | 96 (43.0) | 0.758 (0.469-1.224) | NR | 0.606 (0.403-0.911) | 0.727 (0.407-1.297) | 0.538 (0.367-0.789) | 0.946 (0.487-1.840) | 1.075 (0.596-1.940) | 0.466 (0.311-0.697) | NR | NR | NR | NR | 0.370 (0.153-0.898) | 0.729 (0.505-1.052) | NR | NR |
|  | CT | 36 (32.4) |  | NR | 27 (24.3) |  | NR | 63(57) |  | NR | 48 (43.2) |  | NR |  |  |  |  |  |  | NR | NR | NR | NR |  |  | NR | NR |
| Wang (2021) | TisleplusCT | 42 (35.0) | 0.50 (0.28-0.89) | NR | 30 (25.0) | 0.44 (0.22-0.87) | NR | 72(60) | 0.64 (0.37-1.10) | NR | 48 (40.0) | 0.64 (0.37-1.10) | NR | 0.47 (0.31-0.70) | 0.60 (0.31-1.18) | 0.53 (0.37-0.76) | 0.53 (0.17-1.61) | 0.48 (0.23-1.00) | 0.53 (0.36-0.79) | NR | NR | NR | NR | 0.48 (0.19-1.22) | 0.51 (0.35-0.73) | NR | NR |
|  | CT | 41 (33.9) |  | NR | 31 (25.6) |  | NR | 72(59.5) |  | NR | 49 (40.5) |  | NR |  |  |  |  |  |  | NR | NR | NR | NR |  |  | NR | NR |
| Wang (2021)1 | TisleplusCT | 42 (35.3) | 0.43 (0.23-0.78) | NR | 30 (25.2) | 0.31 (0.15-0.66) | NR | 72(60.5) | 0.37 (0.23-0.59) | NR | 47 (39.5) | 0.69 (0.41-1.18) | NR | 0.47 (0.31-0.72) | 0.56 (0.30-1.05) | 0.50 (0.35-0.71) | 0.36 (0.09-1.47) | 0.12 (0.03-0.53) | 0.56 (0.38-0.80) | NR | NR | NR | NR | 0.48 (0.19-1.19) | 0.46 (0.31-0.67) | NR | NR |
|  | CT | 41 (33.9) |  | NR | 31 (25.6) |  | NR | 72(59.5) |  | NR | 49 (40.5) |  | NR |  |  |  |  |  |  | NR | NR | NR | NR |  |  | NR | NR |
| Zhang (2022) | SintiplusCT | 107 (40) | 0.310 (0.197-0.489) | NR | 74 (28) | 0.503 (0.276-0.918) | NR | 181 (68) | 0.387 (0.272, 0.550) | 0.56 (0.40-0.77) | 85 (32) | 0.664 (0.406-1.086) | 0.75 (0.48-1.19) | PFS:0.432 (0.279-0.668) | 0.526 (0.358- 0.774) | 0.443 (0.320-0.612) | 0.603 (0.332-1.098) | 0.558 (0.337-0.924) | 0.446 (0.315-0.634) | NR | NR | 0.578 (0.283-1.180) | 0.468 (0.342-0.641) | NR | NR | 0.525  (0.308, 0.896) | 0.459 (0.328, 0.643) |
|  | CT | 61 (47) |  | NR | 26 (20) |  | NR | 87 (66) |  |  | 44 (34) |  |  | OS:0.59 (0.39-0.88) | 0.71 (0.50-1.01) | 0.57 (0.43-0.77) | 0.99 (0.56-1.77) | 0.91 (0.57-1.45) | 0.56 (0.41-0.77) | NR | NR | NR | NR | NR | NR | 0.53 (0.32, 0.87) | 0.70 (0.51, 0.95) |
| Zhou (2021) | SintiplusCT | 58 (32.4) | 0.458 (0.302-0.695) | NR | 62 (34.6) | 0.620 (0.408-0.941) | NR | 120 (67.0) | 0.526 (0.392-0.704) | NR | 59 (33.0) | 0.548 (0.368-0.815) | NR | PFS:0.485(0.330-0.712) | 0.572 (0.421-0.778) | 0.532 (0.414 -0.682) | 0.583 (0.252-1.348) | 0.614 (0.333-1.132) | 0.522 (0.403-0.676) | NR | NR | NR | NR | 0.619 (0.271-1.414) | 0.529 (0.412-0.679) | 0.591 (0.405-0.863) | 0.499 (0.369-0.675) |
|  | CT | 63 (35.4) |  | NR | 52 (29.2) |  | NR | 115 (64.6) |  | NR | 63 (35.4) |  | NR | NR | NR | NR | NR | NR | NR | NR | NR | NR | NR | NR | NR | NR | NR |
| Wang (2022) | ToripplusCT | NR | 0.45 (0.27-0.78) | 0.82 (0.39-1.90) | NR | 0.56 (0.40-0.78) | 0.72 (0.48-1.07) | 201 (65.0) | NR | NR | 108 (35.0) | 0.47 (0.32-0.71) | 0.79 (0.49-1.31) | PFS:0.51 (0.38-0.68) | 0.53 (0.368-0.762) | 0.5 (0.39-0.64) | 0.58 (0.35-1.00) | 0.50 (0.33-0.76) | 0.48 (0.37-0.64) | 0.48 (0.35-0.66) | 0.49 (0.35-0.69) | NR | NR | 0.64 (0.31-1.36) | NR | NR | NR |
|  | CT | NR |  |  | NR |  |  | 103 (66.0) | NR | NR | 53 (34.0) |  |  | OS: 0.57 (0.39-0.82) | 0.89 (0.58-1.40) | 0.73 (0.54-0.98) | 0.61 (0.30-1.30) | 0.67 (0.39-1.17) | 0.70 (0.51-0.98) | 0.48 (0.32-0.71) | 0.99 (0.67-1.48) | NR | NR | 1.05 (0.50-2.35) | NR | NR | NR |
| Govindan (2017) | IpiplusCT | NR | NR | NR | NR | NR | NR | NR | NR | NR | NR | NR | NR | NR | NR | NR | NR | NR | NR | NR | NR | NR | NR | NR | NR | NR | NR |
|  | CT | NR | NR | NR | NR | NR | NR | NR | NR | NR | NR | NR | NR | OS:0.82 (0.64-1.04) | 1.06 (0.81-1.37) | 0.85 (0.71-1.02) | 1.33 (0.84-2.11) | 1.19 (0.71-1.99) | 0.88 (0.73-1.05) | NR | NR | NR | NR | NR | NR | NR | NR |
| Ares (2021) | NivoplusIpiplusCT | 68(18.9) | NR | 0.66 (0.44-0.99) | 55(15.2) | NR | 0.61 (0.44-0.84) | 57(15.8) | NR | 0.64 (0.50-0.82) | 64(17.7) | NR | 0.62 (0.45-0.85) | NR | NR | NR | NR | NR | NR | NR | NR | NR | NR | NR | NR | NR | NR |
|  | CT | 46(12.9) | NR |  | 37(10.4) | NR |  | 39(10.9) | NR |  | 35(9.8) | NR |  | OS:0.61 (0.47-0.80) | 0.62 (0.46-0.85) | 0.66 (0.53-0.82) | 0.68 (0.47-1.00) | 1.14 (0.66-1.97) | 0.62 (0.50-0.75) | 0.69 (0.55-0.87) | 0.62 (0.45-0.86) | 0.38 (0.24-0.60) | 0.75 (0.61-0.92) | 0.83 (0.57-1.20) | 0.64 (0.51-0.80) | NR | NR |
| Johnson (2022) | DurvaplusTremeplusCT | 101 (29.9) | NR | 0.65 (0.47-0.89) | 237 (70.1) | NR | 0.82 (0.67-1.00) | 213 (63.0) | NR | 0.76 (0.61-0.95) | 125 (37.0) | NR | 0.77 (0.58-1.00) | NR | NR | NR | NR | NR | NR | NR | NR | NR | NR | NR | NR | NR | NR |
|  | CT | 97 (28.8) | NR |  | 240 (71.2) | NR |  | 207 (61.4) | NR |  | 130 (38.6) | NR |  | OS:0.79 (0.62-1.00) | 0.74 (0.58-0.94) | 0.70 (0.58-0.85) | 0.96 (0.66-1.38) | 1.15 (0.79-1.67) | 0.54 (0.37-0.79) | 0.70 (0.56-0.87) | 0.88 (0.68-1.16) | NR | NR | NR | NR | NR | NR |
| Johnson (2022)1 | DurvaplusCT | 94 (27.8) | NR | 0.63 (0.45-0.88) | 243 (71.9) | NR | 0.94 (0.77-1.14) | 224 (66.3) | NR | 0.79 (0.64-0.98) | 113 (33.4) | NR | 0.99 (0.76-1.30) | NR | NR | NR | NR | NR | NR | NR | NR | NR | NR | NR | NR | NR | NR |
|  | CT | 97 (28.8) | NR |  | 240 (71.2) | NR |  | 207 (61.4) | NR |  | 130 (38.6) | NR |  | OS:0.86 (0.68-1.10) | 0.81 (0.64-1.03) | 0.81 (0.67-0.98) | 0.91 (0.64-1.30) | 0.92 (0.65-1.31) | 0.77 (0.52-1.12) | 0.82 (0.66-1.03) | 0.84 (0.64-1.10) | NR | NR | NR | NR | NR | NR |

**Table S2** Grade ≥3 treatment-related adverse events.

| **Study** | **Medicine** | **E/C** | **Anemia** | **Neutrophil count decreased** | **Platelet count decreased** | **Pneumonitis** | **White blood cell count decreased** | **Fatigue** | **Nausea** |
| --- | --- | --- | --- | --- | --- | --- | --- | --- | --- |
| KEYNOTE-021 | pembrolizumab+CT | 60 | 20 (34%) | 10 (17%) | NR | **4 (7%)** | NR | 40 (68%) | 35 (59%) |
|  | CT | 63 | 35 (56%) | 8 (13%) | NR | **0** | NR | 28 (45%) | 30 (48%) |
| GEMSTONE-302 | sugemalimab+CT | 320 | 43 (13%) | 104（33%） | 33（11%） | 6（2%） | 45（14%） | 3 (1%) | 1 (<1%) |
|  | CT | 159 | 18 (12%) | 52(33%) | 15(10%) | 6（4%） | 27(17%) | 1 (1%) | 3 (2%) |
| CameL | camrelizumab+CT | 205 | 38 (19%) | 78 (38%) | 34 (17%) | 4 (2%) | 40 (20%) | 7 (3%) | 2 (<1%) |
|  | CT | 207 | 23 (11%) | 63 (30%) | 24 (12%) | 1 (<1%) | 30 (14%) | 3 (1%) | 2 (<1%) |
| CameL-sq | camrelizumab+CT | 193 | 20 (10%) | 107 (55%) | 13 (7%) | 7 (4%) | 58 (30%) | 3 (1.6) | 0 |
|  | CT | 196 | 14 (7%) | 116 (59%) | 7 (4%) | 9 (5%) | 51 (26%) | 3 (2%) | 1 (<1%) |
| KEYNOTE-189 | pembrolizumab+CT | 410 | 75 (18.5%) | 66 (16.3%) | 34 (8.4%) | 12 (3.0%) | NR | 31 (7.7%) | 14 (3.5%) |
|  | CT | 206 | 35 (17.3%) | 24 (11.9%) | 15 (7.4%) | 4 (2.0%) | NR | 7 (3.5%) | 8 (4.0%) |
| IMpower-132 | atezolizumab+CT | 292 | NR | NR | NR | 6 (2.1) | NR | NR | NR |
|  | CT | 286 | NR | NR | NR | 3 (1.1) | NR | NR | NR |
| KEYNOTE-407 | pembrolizumab+CT | 278 | 44 (15.8%) | 64 (23.0%) | 23 (8.3%) | 9 (3.2%) | NR | 13 (4.7%) | 4 (1.4%) |
|  | CT | 281 | 58 (20.7%) | 69 (24.6%) | 19 (6.8%) | 3 (1.1%) | NR | 12 (4.3%) | 3 (1.1%) |
| RATIONALE-304 | tislelizumab+CT | 222 | 33 (14.9%) | 99 (44.6%) | 43 (19.4%) | NR | 48 (21.6%) | 3 (1.4%) | 1 (0.5%) |
|  | CT | 110 | 13 (11.8%) | 39 (35.5%) | 15 (13.6%) | NR | 16 (14.5%) | 2 (1.8%) | 1 (0.9%) |
| RATIONALE-307 | tislelizumab+CT | 120 | 9 (7.5%) | 62 (51.7%) | 5 (4.2%) | NR | 27 (22.5%) | 0 | 0 |
|  | tislelizumab+CT | 119 | 27 (22.9%) | 54 (45.8%) | 16 (13.6%) | NR | 32 (27.1%) | 0 | 0 |
|  | CT | 121 | 14 (12.0%) | 53 (45.3%) | 2 (1.7%) | NR | 28 (23.9%) | 1 (0.9%) | 1 (0.9%) |
| ORIENT-11 | sintilimab+CT | 266 | 40 (15.0%) | 97 (36.5%) | 32 (12.0%) | 2 (0.8%) | 39 (14.7%) | 2 (0.8%) | 4 (1.5%) |
|  | CT | 131 | 25 (19.1%) | 40 (30.5%) | 16 (12.2%) | 1 (0.8%) | 20 (15.3%) | 2 (1.5%) | 0 |
| ORIENT-12 | sintilimab+CT | 179 | 60 (33.5%) | 87 (48.6%) | 81 (45.3%) | 25 (14.0%) | 65 (36.3%) | 3 (1.7%) | 2 (1.1%) |
|  | CT | 178 | 57 (32.0%) | 85 (47.8%) | 76 (42.7%) | 17 (9.6%) | 65 (36.5%) | 2 (1.1%) | 1 (0.6%) |
| CHOICE-01 | toripalimab+CT | 309 | 92（29.9%) | 171(55.5%) | 53(17.2%) | 10（3.2%) | 110(35.7%) | 4（1.3%) | 1（0.3%) |
|  | CT | 156 | 56(35.9%) | 84(53.8%) | 28(17.9%) | 0 | 65(41.7%) | 3（1.9%) | 0 |
| Lynch et al.2012 | ipilimumab+CT followed by CT | 70 | 8（11%) | 6（8%) | 1（2%) | NR | NR | 6（8%) | 1（1%) |
|  | CT followed by ipilimumab+CT | 68 | 4（6%) | 2（3%) | 2（3%) | NR | NR | 3（5%) | 1（2%) |
|  | CT | 66 | 4（6%) | 7（10%) | 7（10%) | NR | NR | 3（5%) | 1（2%) |
| Govindan R, NCT01285609 | ipilimumab+CT | 388 | 46（12%) | 55（14%) | 28（7%) | NR | 10（3%) | 12（3%) | 1（1%) |
|  | CT | 361 | 27（7%) | 50（14%) | 13（4%) | NR | 10（3%) | 10（3%) | 0 |
| CheckMate 9LA | nivolumab+ipilimumab+CT | 361 | 21(6%) | 24（7%） | 10（2%） | NR | 5 (1%) | 8 (2%) | 5 (1%) |
|  | CT | 358 | 50 (14%) | 32 (9%) | 9（3%） | NR | 2 (1%) | 2 (1%) | 3 (1%) |
| POSEIDON | durvalumab+tremelimumab+CT | 338 | 57 (17.3%) | 53 (16.1%) | 18 (5.5%) | 3 (0.9%) | 9 (2.7%) | 5 (1.5%) | 4 (1.2%) |
|  | durvalumab+CT | 338 | 51 (15.3%) | 42 (12.6%) | 15 (4.5%) | 4 (1.2%) | 8 (2.4%) | 7 (2.1%) | 1 (0.3%) |
|  | CT | 337 | 68 (20.4%) | 40 (12.0%) | 17 (5.1%) | 2 (0.6%) | 12 (3.6%) | 7 (2.1%) | 5 (1.5%) |
| KEYNOTE-189 Japan Study | pembrolizumab+CT | 25 | 2 (8%) | 5 (20%) | 0 | 1 (4%) | 4 (16%) | 0 | 0 |
|  | CT | 15 | 3 (20%) | 0 | 0 | 2 (13%) | 1 (7%) | 0 | 1 (7%) |

**Table S3** League chart of ORR.

| AtezoplusCT | 0.85 (0.53, 1.35) | 1.46 (1, 2.13) | 0.86 (0.5, 1.47) | 0.92 (0.53, 1.58) | 1.41 (0.8, 2.14) | 0.98 (0.57, 1.67) | 0.79 (0.5, 1.22) | 0.99 (0.61, 1.59) | 0.98 (0.57, 1.67) | 0.97 (0.62, 1.51) | 1.02 (0.6, 1.73) |
| --- | --- | --- | --- | --- | --- | --- | --- | --- | --- | --- | --- |
| 1.18 (0.74, 1.88) | CamreplusCT | 1.72 (1.31, 2.27) | 1.01 (0.63, 1.63) | 1.08 (0.67, 1.74) | 1.67 (1.03, 2.35) | 1.15 (0.72, 1.85) | 0.93 (0.65, 1.32) | 1.17 (0.78, 1.73) | 1.16 (0.73, 1.84) | 1.15 (0.8, 1.64) | 1.21 (0.76, 1.92) |
| 0.68 (0.47, 1) | 0.58 (0.44, 0.76) | CT | 0.59 (0.4, 0.86) | 0.63 (0.42, 0.93) | 0.97 (0.66, 1.23) | 0.67 (0.45, 0.98) | 0.54 (0.42, 0.67) | 0.68 (0.5, 0.9) | 0.67 (0.46, 0.98) | 0.67 (0.53, 0.83) | 0.7 (0.48, 1.01) |
| 1.17 (0.68, 2.01) | 0.99 (0.62, 1.59) | 1.71 (1.16, 2.52) | DurvaplusCT | 1.07 (0.62, 1.86) | 1.65 (0.93, 2.53) | 1.14 (0.66, 1.97) | 0.92 (0.58, 1.43) | 1.16 (0.71, 1.87) | 1.15 (0.66, 1.96) | 1.14 (0.72, 1.78) | 1.2 (0.69, 2.04) |
| 1.09 (0.63, 1.88) | 0.92 (0.58, 1.49) | 1.59 (1.08, 2.36) | 0.93 (0.54, 1.62) | DurvaplusTremeplusCT | 1.54 (0.87, 2.37) | 1.07 (0.61, 1.85) | 0.86 (0.54, 1.34) | 1.08 (0.66, 1.75) | 1.07 (0.62, 1.83) | 1.06 (0.68, 1.66) | 1.11 (0.65, 1.9) |
| 0.71 (0.47, 1.25) | 0.6 (0.43, 0.97) | 1.03 (0.81, 1.51) | 0.61 (0.4, 1.07) | 0.65 (0.42, 1.15) | IpiplusCT | 0.69 (0.45, 1.22) | 0.56 (0.4, 0.87) | 0.7 (0.49, 1.15) | 0.69 (0.46, 1.22) | 0.69 (0.5, 1.08) | 0.72 (0.48, 1.26) |
| 1.02 (0.6, 1.76) | 0.87 (0.54, 1.4) | 1.49 (1.02, 2.21) | 0.88 (0.51, 1.52) | 0.94 (0.54, 1.63) | 1.45 (0.82, 2.21) | NivoplusIpiplusCT | 0.81 (0.51, 1.26) | 1.01 (0.62, 1.64) | 1 (0.59, 1.72) | 1 (0.64, 1.56) | 1.05 (0.61, 1.79) |
| 1.27 (0.82, 1.99) | 1.08 (0.76, 1.55) | 1.85 (1.49, 2.35) | 1.09 (0.7, 1.72) | 1.16 (0.75, 1.85) | 1.79 (1.15, 2.48) | 1.24 (0.8, 1.95) | PembroplusCT | 1.26 (0.87, 1.82) | 1.25 (0.81, 1.95) | 1.24 (0.9, 1.72) | 1.3 (0.84, 2.02) |
| 1.01 (0.63, 1.64) | 0.86 (0.58, 1.28) | 1.48 (1.11, 1.99) | 0.87 (0.54, 1.41) | 0.93 (0.57, 1.51) | 1.43 (0.87, 2.05) | 0.99 (0.61, 1.61) | 0.8 (0.55, 1.15) | SintiplusCT | 0.99 (0.62, 1.6) | 0.98 (0.68, 1.43) | 1.03 (0.64, 1.66) |
| 1.02 (0.6, 1.75) | 0.86 (0.54, 1.37) | 1.49 (1.02, 2.18) | 0.87 (0.51, 1.5) | 0.93 (0.55, 1.6) | 1.44 (0.82, 2.18) | 1 (0.58, 1.71) | 0.8 (0.51, 1.24) | 1.01 (0.62, 1.62) | SugeplusCT | 0.99 (0.64, 1.54) | 1.04 (0.61, 1.77) |
| 1.03 (0.66, 1.6) | 0.87 (0.61, 1.24) | 1.5 (1.2, 1.89) | 0.88 (0.56, 1.38) | 0.94 (0.6, 1.48) | 1.45 (0.92, 1.98) | 1 (0.64, 1.57) | 0.81 (0.58, 1.11) | 1.02 (0.7, 1.47) | 1.01 (0.65, 1.56) | TisleplusCT | 1.05 (0.68, 1.62) |
| 0.98 (0.58, 1.68) | 0.83 (0.52, 1.32) | 1.43 (0.99, 2.09) | 0.84 (0.49, 1.44) | 0.9 (0.53, 1.54) | 1.38 (0.79, 2.09) | 0.96 (0.56, 1.63) | 0.77 (0.49, 1.19) | 0.97 (0.6, 1.56) | 0.96 (0.57, 1.63) | 0.95 (0.62, 1.48) | ToripplusCT |

**Table S4** League chart of DCR.

| CamreplusCT | 1.07 (0.96, 1.22) | 1.16 (0.99, 1.38) | 0.98 (0.81, 1.21) | 0.91 (0.79, 1.07) | 0.93 (0.75, 1.16) | 0.96 (0.83, 1.14) |
| --- | --- | --- | --- | --- | --- | --- |
| 0.93 (0.82, 1.04) | CT | 1.08 (0.96, 1.21) | 0.91 (0.78, 1.08) | 0.85 (0.77, 0.94) | 0.87 (0.72, 1.03) | 0.9 (0.81, 1) |
| 0.86 (0.72, 1.01) | 0.92 (0.82, 1.04) | IpiplusCT | 0.85 (0.69, 1.04) | 0.79 (0.67, 0.92) | 0.8 (0.65, 0.99) | 0.83 (0.71, 0.97) |
| 1.02 (0.83, 1.24) | 1.09 (0.93, 1.29) | 1.18 (0.97, 1.45) | NivoplusIpiplusCT | 0.93 (0.77, 1.13) | 0.95 (0.74, 1.2) | 0.98 (0.81, 1.19) |
| 1.09 (0.93, 1.27) | 1.18 (1.06, 1.3) | 1.27 (1.09, 1.48) | 1.07 (0.89, 1.3) | PembroplusCT | 1.02 (0.83, 1.25) | 1.06 (0.91, 1.22) |
| 1.08 (0.86, 1.32) | 1.15 (0.97, 1.39) | 1.25 (1.01, 1.55) | 1.06 (0.83, 1.35) | 0.98 (0.8, 1.21) | SintiplusCT | 1.04 (0.84, 1.28) |
| 1.04 (0.88, 1.21) | 1.11 (1, 1.24) | 1.2 (1.03, 1.41) | 1.02 (0.84, 1.24) | 0.95 (0.82, 1.1) | 0.96 (0.78, 1.19) | TisleplusCT |
