# Supplementary material for: Comparative efficacy of immune checkpoint inhibitors combined with chemotherapy in patients with advanced driver-gene negative non-small cell lung cancer: A systematic review and network meta-analysis
Source: Heliyon. 2024 May 7;10(10):e30809. doi: 10.1016/j.heliyon.2024.e30809 (PMC11107224; doi:10.1016/j.heliyon.2024.e30809)
Supplement: Multimedia component 3 [file mmc3.docx]

**
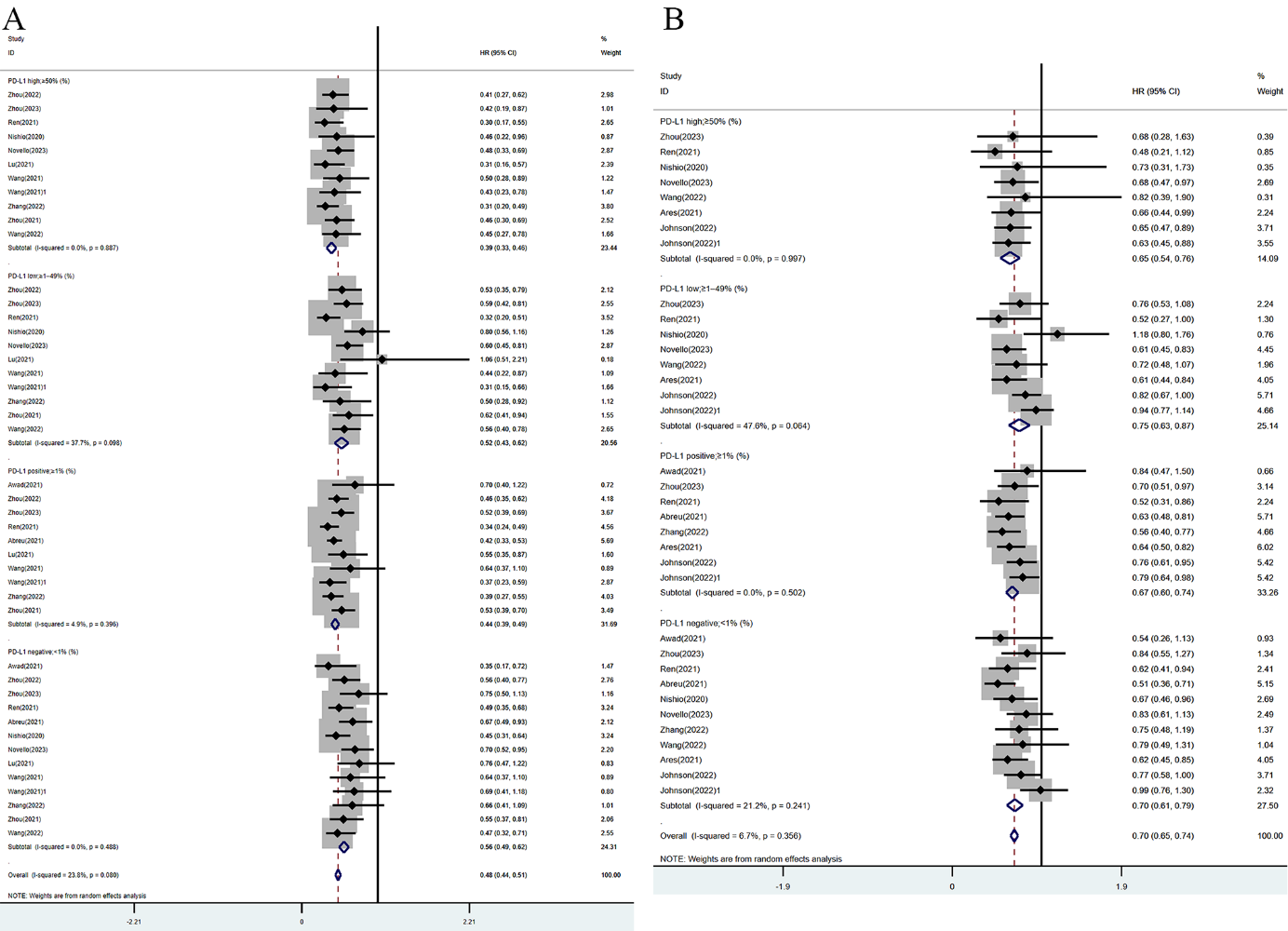
**

**Fig. S1** Subgroup analysis of PFS and OS in patients with different PD-L1 expression levels.

**
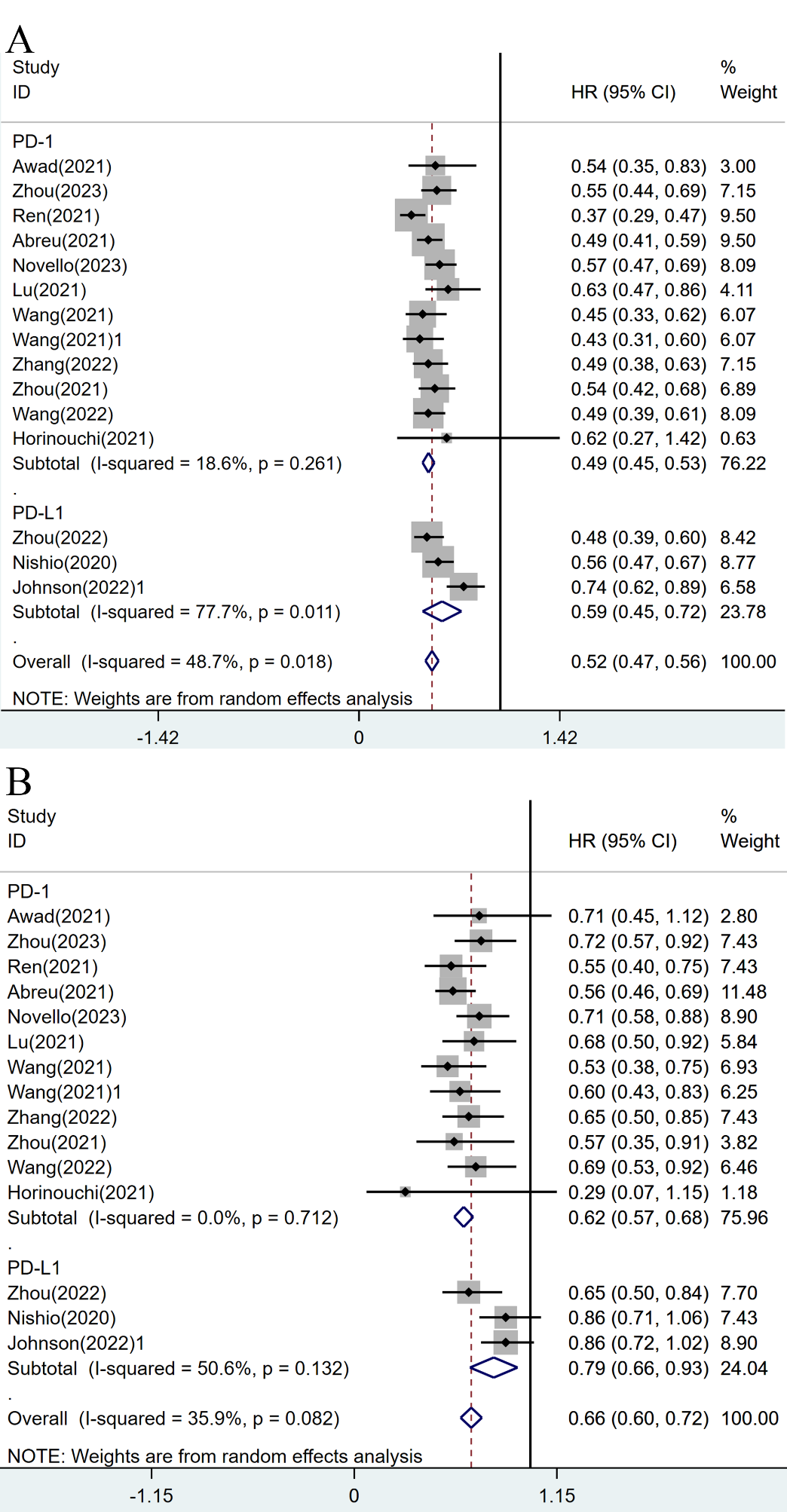
**

**Fig. S2** Subgroup analysis of PFS and OS in patients treated with different ICIs type.

**
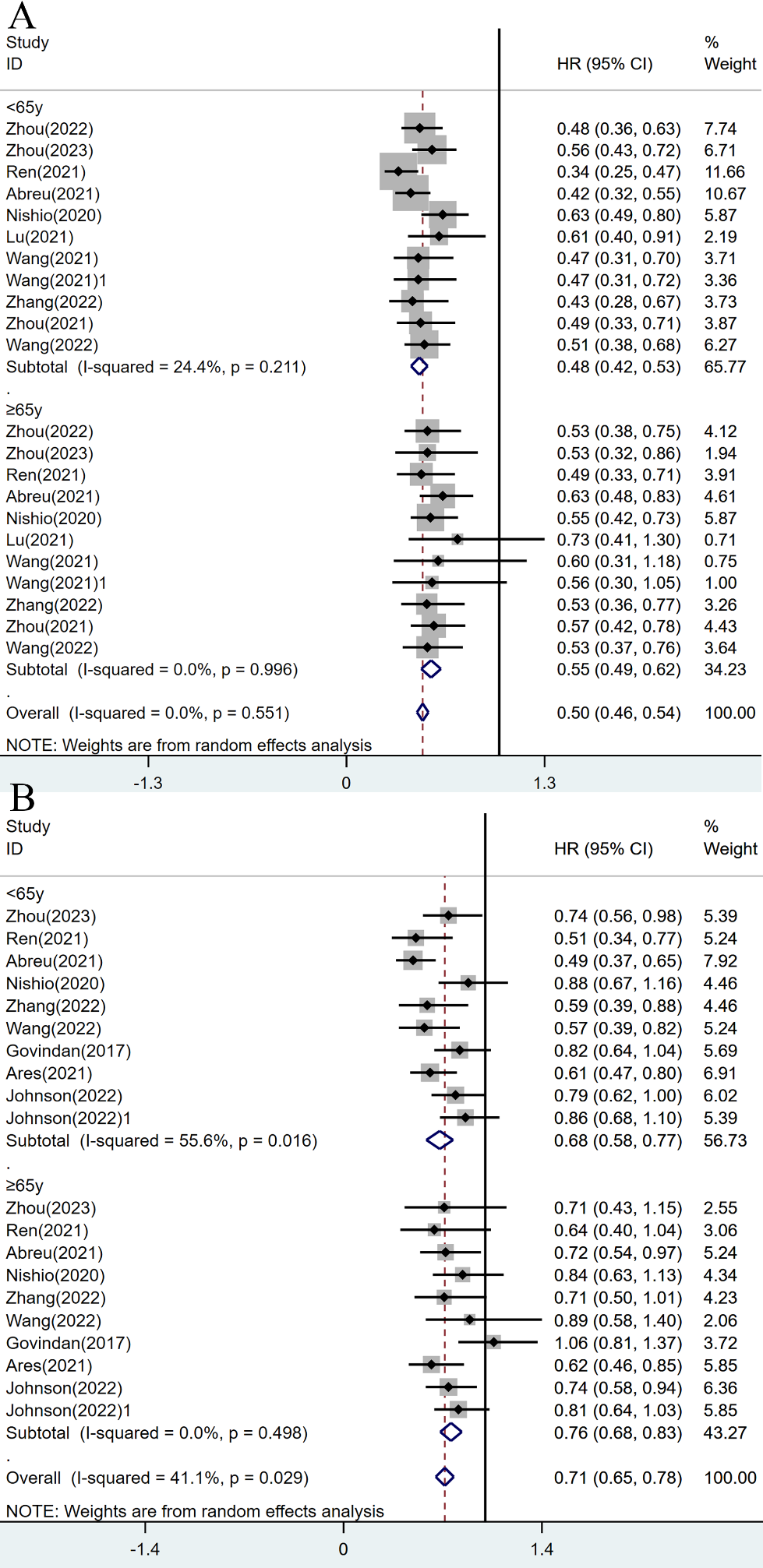
**

**Fig. S3** Subgroup analysis of PFS and OS in patients of different ages.

**
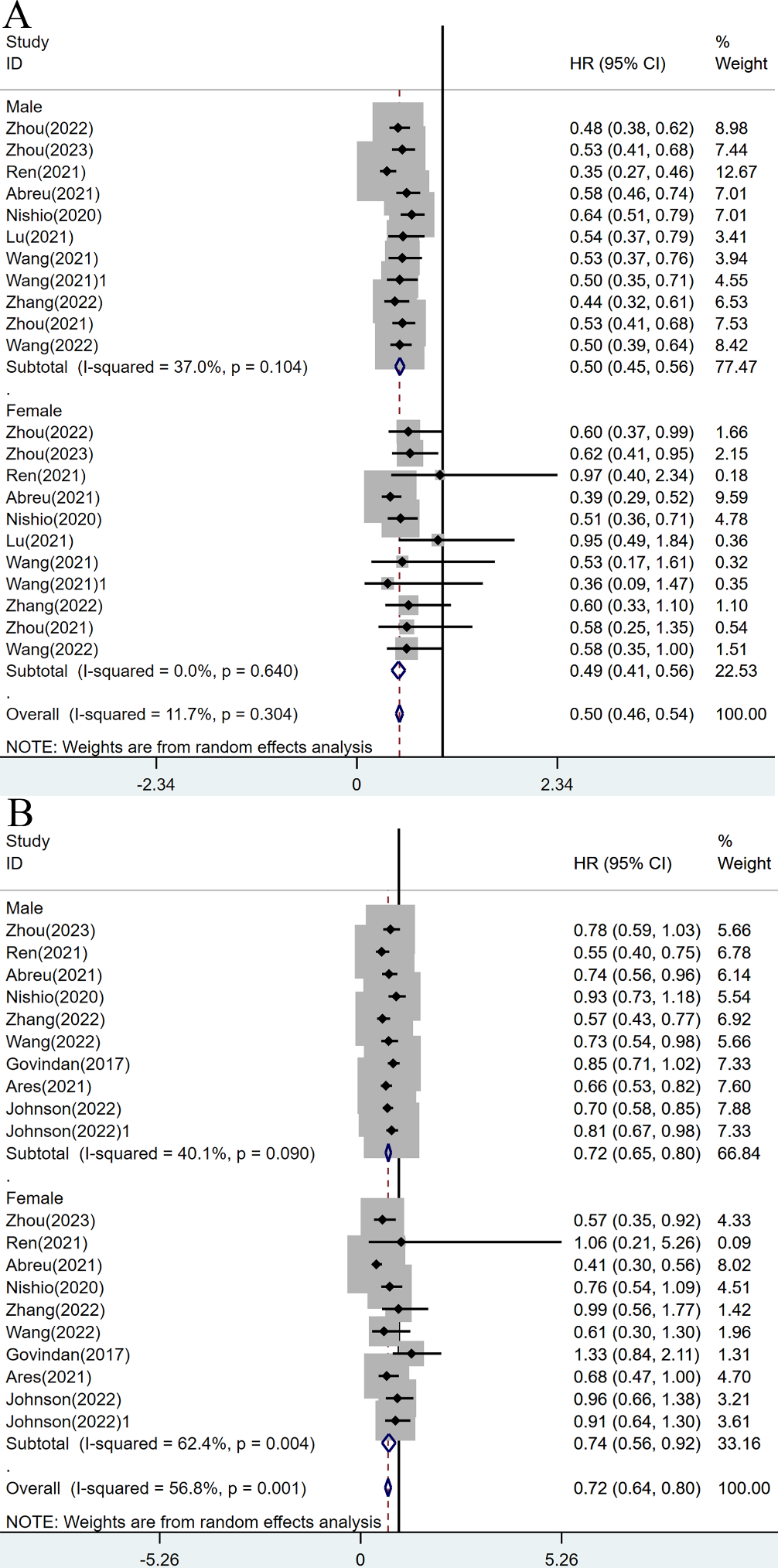
**

**Fig. S4** Subgroup analysis of PFS and OS in Male and Female.

**
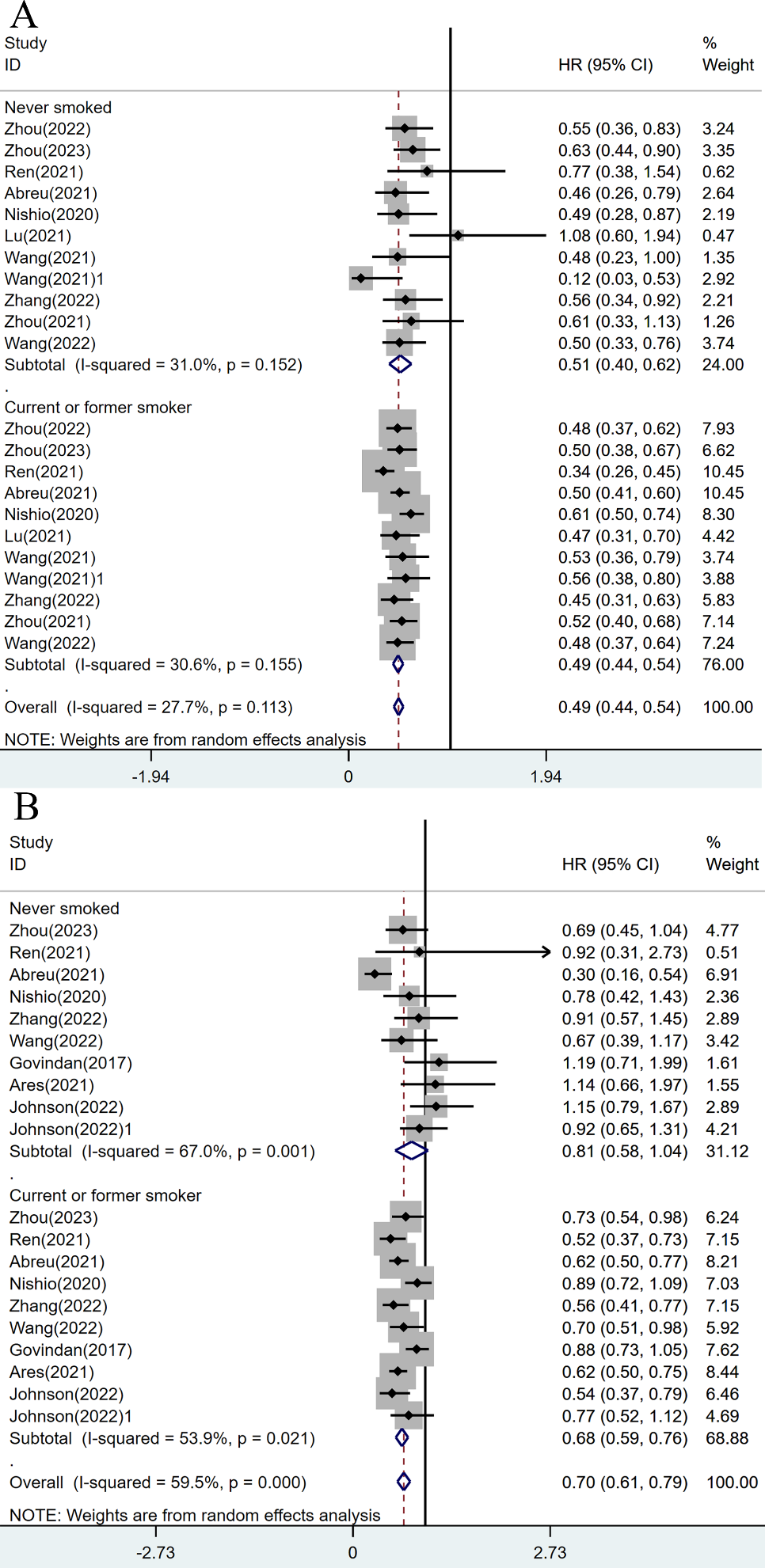
**

**Fig. S5** Subgroup analysis of PFS and OS in Patients with different smoking status.

**
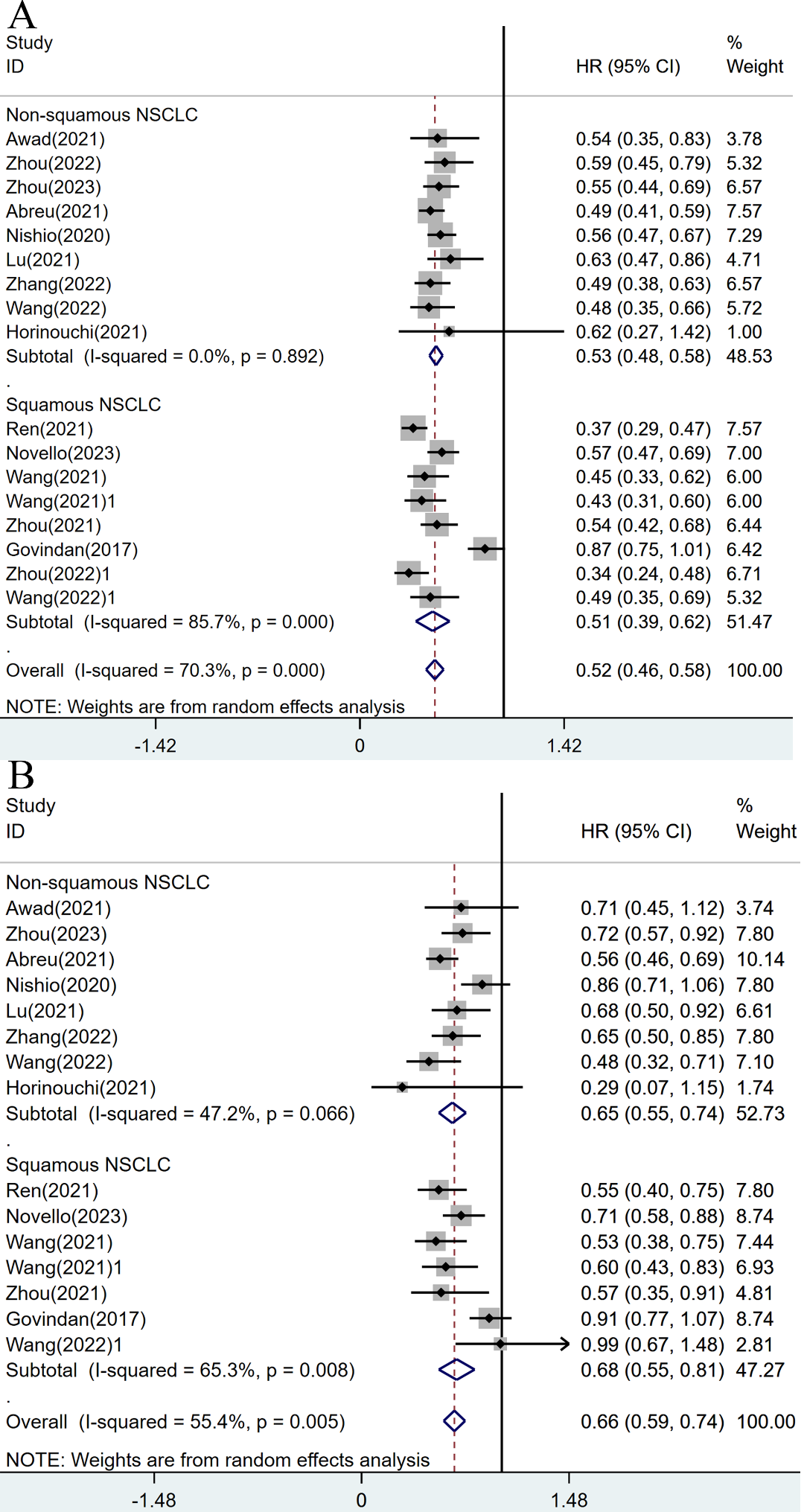
**

**Fig. S6** Subgroup analysis of PFS and OS in Patients with different types of pathology.

**
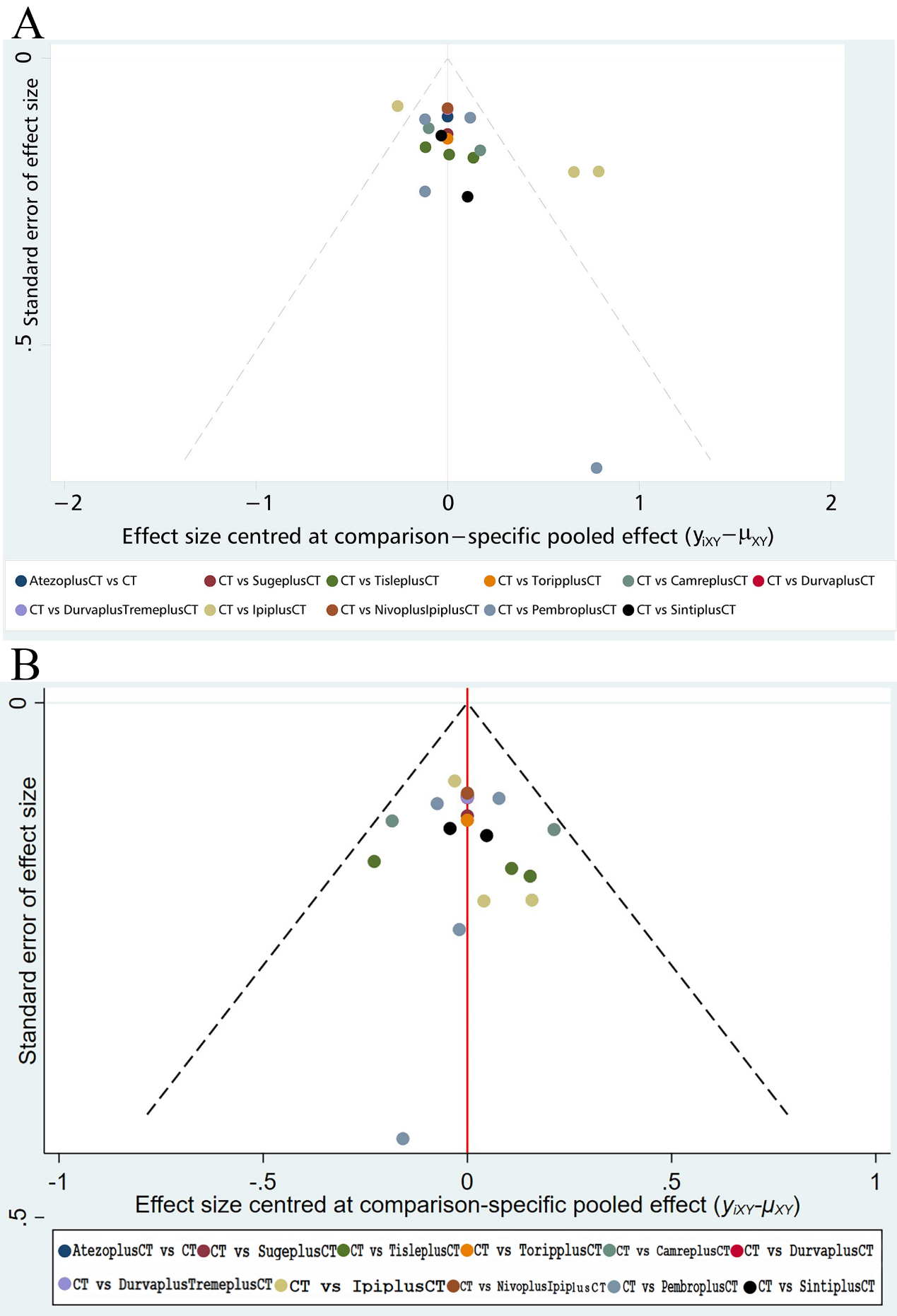
**

**Fig. S7** Funnel plots of different endpoints.

**
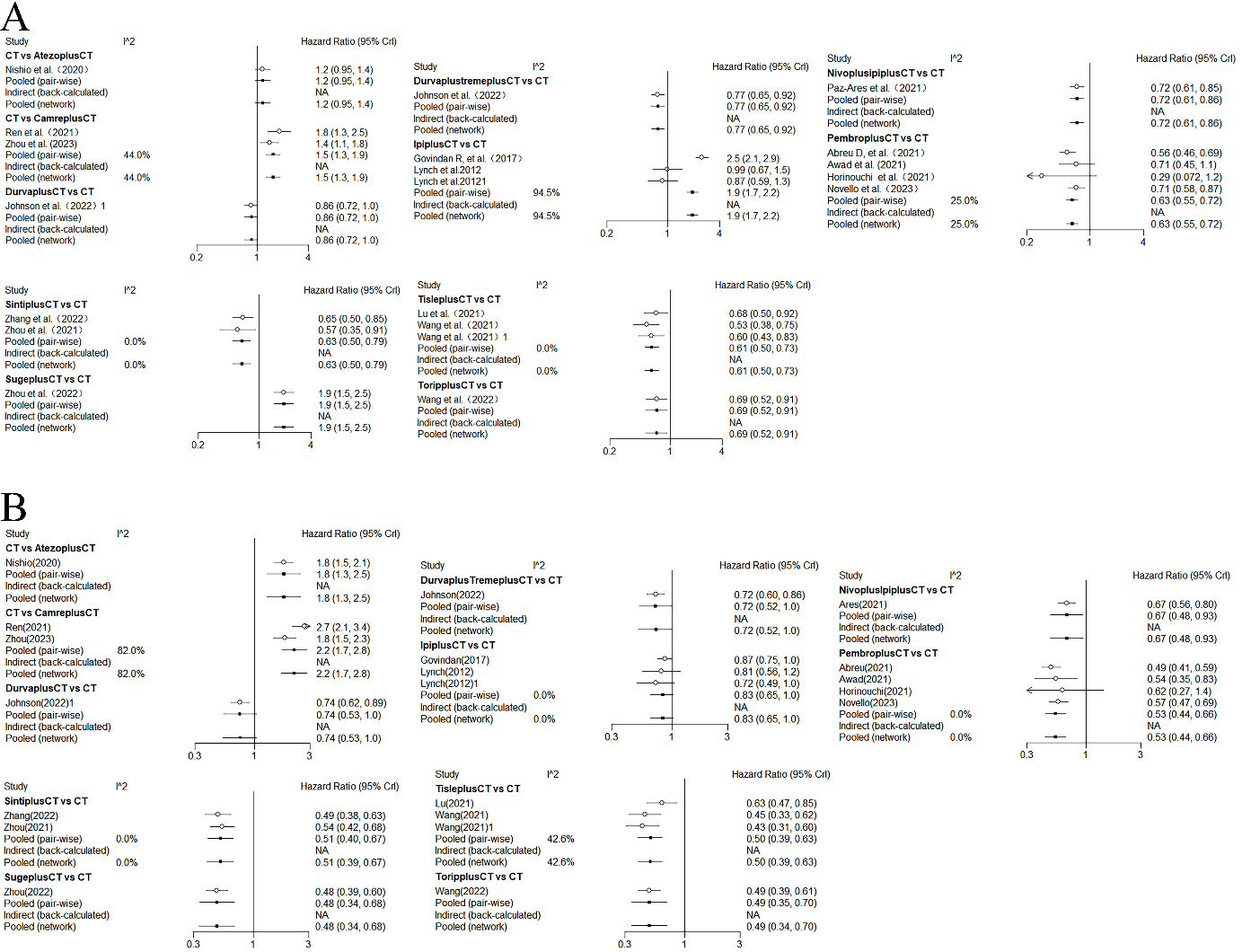
**

**Fig. S8** Heterogeneity Analysis Charts of different endpoints.

**
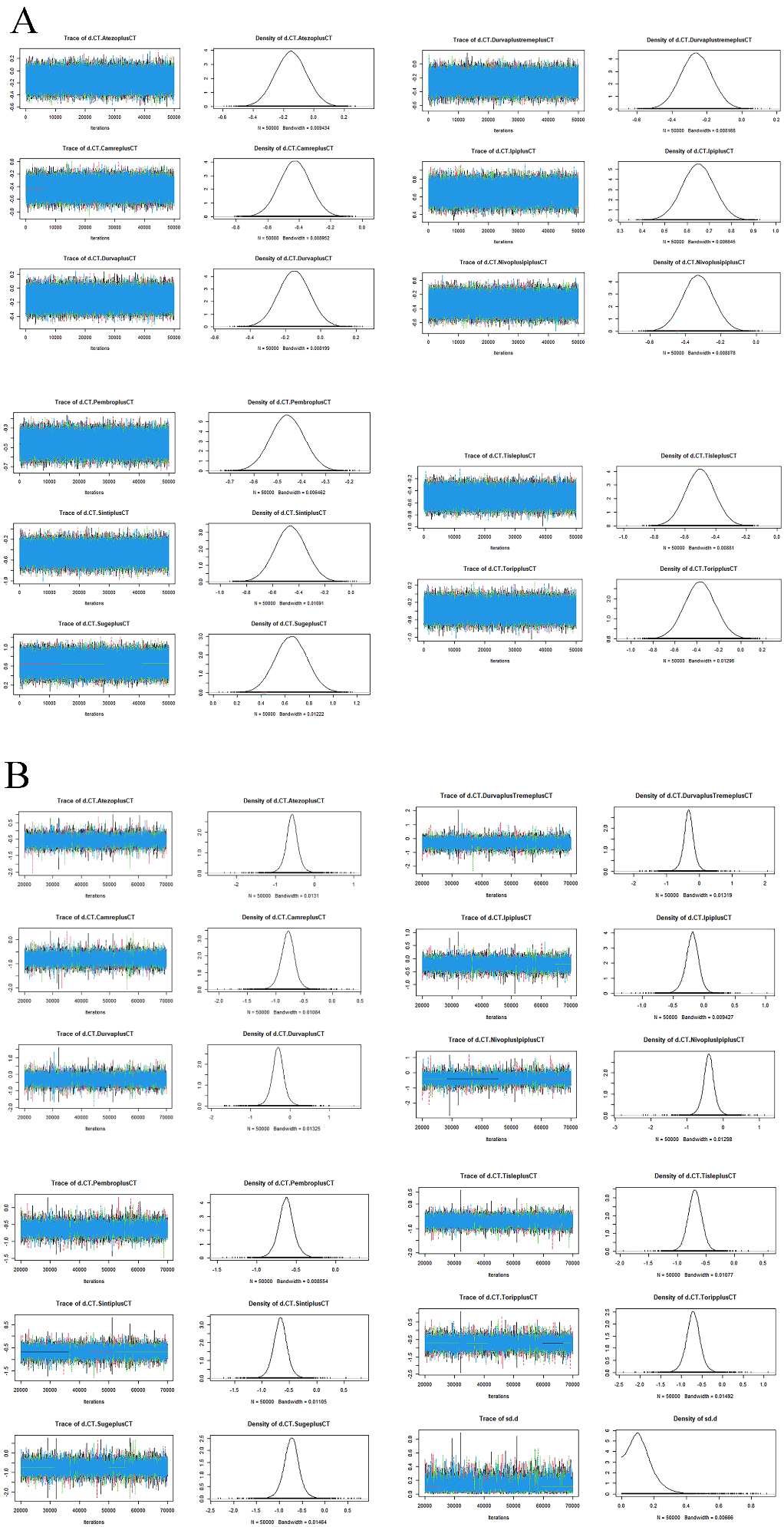
**

**Fig. S9** Density charts of different endpoints.

**
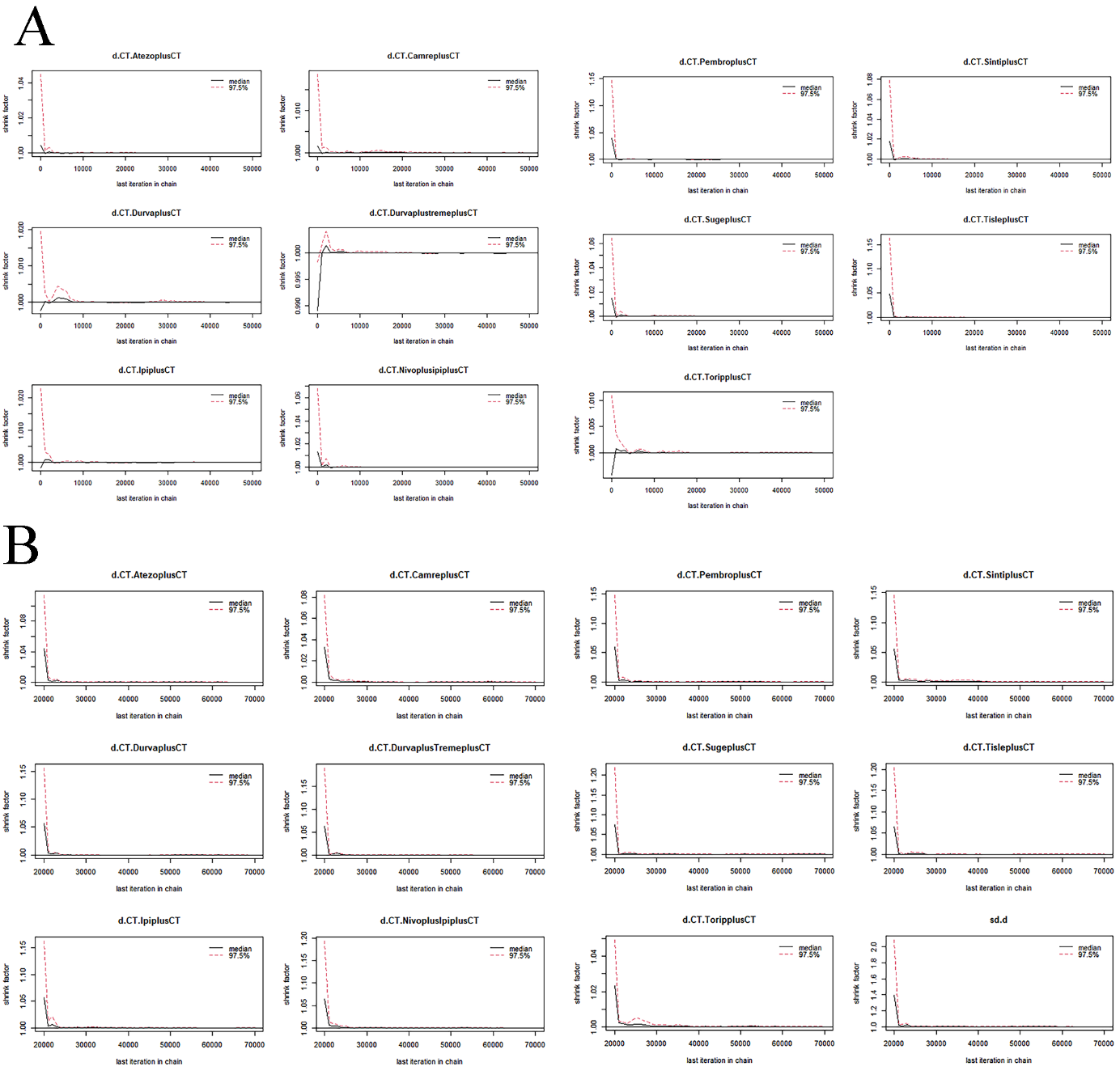
**

**Fig. S10** Convergence charts of different endpoints.
